# Clinical and Biological Significance of DNA Methylation-Driven Differentially Expressed Genes in Biochemical Recurrence After Radical Prostatectomy

**DOI:** 10.3389/fgene.2022.727307

**Published:** 2022-02-02

**Authors:** Chao Luo, Songzhe He, Haibo Zhang, Shuhua He, Huan Qi, Anyang Wei

**Affiliations:** ^1^ Department of Urology, Nanfang Hospital, Southern Medical University, Guangzhou, China; ^2^ Department of Laboratory Medicine, Affiliated Hospital of Guilin Medical University, Guilin, China

**Keywords:** prostate cancer, biochemical recurrence, classifier (classification tool), DNA methylation-driven genes, biomarker

## Abstract

**Background:** Biochemical recurrence (BCR) after radical prostatectomy indicates poor prognosis in patients with prostate cancer (PCA). DNA methylation (DNAm) is a critical factor in tumorigenesis and has attracted attention as a biomarker for the diagnosis, treatment, and prognosis of PCA. However, the predictive value of DNAm-derived differentially expressed genes (DMGs) in PCA with BCR remains elusive.

**Methods:** We filtered the methylated genes and the differentially expressed genes (DGEs) for more than 1,000 clinical samples from the TCGA cohort using the chAMP and DESeq2 packages of R language, respectively. Next, we integrated the DNAm beta value and gene expression data with the Mithymix package of R language to obtain the DMGs. Then, 1,000 times Cox LASSO regression with 10-fold cross validation was performed to screen signature DMGs and establish a predictive classifier. Univariate and multivariate cox regressive analyses were used to identify the prognostic factors to build a predictive model, and its performance was measured by receiver operating characteristic, calibration curves, and Harrell’s concordance index (C-index). Additionally, a GEO dataset was used to validate the prognostic classifier.

**Results:** One hundred DMGs were mined using the chAMP and Methymix packages of R language. Of these, seven DMGs (CCK, CD38, CYP27A1, EID3, HABP2, LRRC4, and LY6G6D) were identified to build the prognostic classifier (Classifier) through LASSO analysis. Moreover, univariate and multivariate Cox regression analysis determined that the Classifier and pathological T stage (pathological_T) were independent predictors of BCR (hazard ratio (HR 2.2), (95% CI 1.4–3.5), *p* < 0.0012, and (HR 1.8), (95% CI 1.0–3.2), *p* < 0.046). A nomogram based on the Classifier was constructed, with high prediction accuracy for BCR-free survival in TCGA and GEO datasets. GSEA enrichment analysis showed that the DMGs were mainly enriched in the metabolism pathways.

**Conclusion:** We identified and validated the nomogram of BCR-free survival for PCA patients, which has the potential to guide treatment decisions for patients at differing risks of BCR. Our study deepens the understanding of DMGs in the pathogenesis of PCA.

## Introduction

Prostate cancer (PCA) is a common cancer with the highest prevalence among men worldwide. In 2018, the global incidence of PCA was 29.3 per 100,000 (WHO, 2018). In the United States, it is estimated that more than 30,000 cases of death in men per year are attributable to PCA (Siegel et al., 2019); furthermore, there are 60.3 new cases of PCA per 100,000 and 26.6 deaths per 100,000 individuals in China (Chen et al., 2016). Radical prostatectomy (RP) is considered as an effective therapy for the treatment of localized PCA. However, up to 20–53% of patients experience biochemical recurrence (BCR) after RP (Mottet et al., 2018). BCR is defined as a serum PSA equal to or greater than 0.2 ng/ml on two consecutive occasions after surgery or radiation. However, some studies and guidelines have indicated that PSA cannot be used to predict BCR for each patient with PCA, especially when its value is very low (Eisenberg et al., 2010; Fendler et al., 2019; Wang et al., 2020). Moreover, patients with similar clinical features or PSA levels might have a different clinical endpoint. Among patients with high-risk PCA and clinical stage ≥ T3a, a biopsy Gleason score of 8–10, and/or a serum PSA level >20 ng/ml, approximately 60% had at least 15 years of metastasis-free survival after RP, indicating that not all patients had poor prognosis (Spahn et al., 2010a; Spahn et al., 2010b). The monitoring of BCR was expected to effectively prevent mortality. However, overtreatment owing to misprediction should also be avoided (Artibani et al., 2018).

Epigenetics and PCA have been studied at great length. The evolution of PCA involves a combination of epigenetic and genetic changes, and methylation is an important mechanism. The methylation of KDM1A and CHD1 genes can drive the transcription and translocation of androgen receptors (Metzger et al., 2016). PCA recurrence can lead to many molecular aberrations, including DNA methylation (DNAm), which can be used as biomarkers of PCA prognosis (Fraser et al., 2017). Additionally, the promoter methylation of CRMP4 in biopsied tissue can predict lymph node metastasis of PCA (Gao et al., 2017). As a critical factor in tumorigenesis, DNAm has attracted increasing attention as a biomarker for the diagnosis, treatment, and prognosis of PCA (Wei et al., 2015). CpG islands are rich in cytosine and guanine dinucleotides and are 200 bp to several kilobases in length. To better regulate highly expressed genes, CpG islands are always in close proximity to the promoters of these genes (Nowacka-Zawisza and Wiśnik, 2017). Additionally, CpG islands can modulate cancer proliferation, including that of PCA, *via* the hypomethylation of cytosines at the 5′position in CpG islands within the promoter region of oncogenes. In contrast, hypermethylation of the regulatory (promoter) region of suppressor genes leads to gene silencing (Herman and Baylin, 2003; Baylin and Ohm, 2006). Alterations of tumors at the molecular level always occur before the manifestation of clinicopathological features (Jordan et al., 2017; Devos et al., 2020). However, to date, no reliable BCR biomarkers for PCA have been identified for routine application in clinical practice.

In this study, we established a practical and reliable nomogram based on DNAm-derived differentially expressed gene (DMG) profiling from The Cancer Genome Atlas (TCGA) data to improve risk stratification for patients with PCA. Moreover, we analyzed Gene Expression Omnibus (GEO) datasets to validate the nomogram and related genes and explored the relationship between methylation status and gene expression. Our findings confirm that these DMGs might be potential therapeutic targets in the future.

## Materials and Methods

### Data Collection

TCGA data (gene expression data, methylation data, and associated clinicopathological features) were downloaded from the Genomic Data Commons (GDC) Data Portal of the National Institutes of Health, and TCGA level-3 molecular data and corresponding clinical data were available through the GDC (up to 2020/4/10; Supplementary Table S1). The DNAm level was measured with β values ranging from 0 to 1 (the Illumina Infinium Human Methylation 450 platform of the GDC). Furthermore, the inclusion criteria for the discovery cohort (TCGA cohort) were as follows: 1) patients who had undergone RP; 2) patients with associated clinicopathological features, such as BCR time, BCR status, residual tumor data, TNM stage, lymph node number, pathologic Gleason Score, target therapy, radiotherapy, and laterality; and 3) clinical results assessed using BCR time. For the non-BCR samples without BCR time, their last follow-up time was used for further study.

### Identification of Differentially Expressed Genes

After downloading raw RNA-sequencing datasets of TCGA prostate adenocarcinoma (PRAD) cohorts (HTSeq-Counts of TCGA-PRAD transcriptome profiling) and deleting the duplicated samples, we extracted DEGs between 474 PCA and 53 nontumorous tissues using the “DESeq2”, package (Love et al., 2014). Here, for multiple probes, the average value corresponding to the same gene is taken during the calculation. An absolute logFC >1 and false discovery rate (FDR) < 0.05 were set as the cut-off values. The results were visualized using the R language package “ggplot2.”

### Filtering and Cleaning of Methylation Data

The DNAm data contained the 499 PCA and 50 nontumorous tissues. The data were filtered using the chAMP package of R language (Phipson et al., 2016) according to the following criteria: 1) filter out probes with a *p*-value greater than 0.01; 2) filter out probes with a bead count less than 3 in at least 5% of the samples; and 3) filter out probes at non-CpG sites; 4) filter all SNP-related probes (R code: Supplementary File S1). In case of multiple CpG sites being annotated by one methylated gene, we could calculate their average value using the “aggregate function”of R language. The CpGs annotation file was obtained from the TCGA dataset.

### Identification of DMGs

DMGs were identified by integrating the methylated genes and DEGs with the “MethylMix” package. A new version of MethylMix was developed to automatically preprocess the databases of methylation-driven genes and subsequently analyze their transcriptionally predictive methylation states by applying the MethylMix algorithm (Gevaert, 2015). First, a correlation analysis was performed between the gene expression data of DEGs in the PCA samples and their corresponding methylation data. The target genes with a correlation coefficient < −0.3 and *p*-value <0.05 were used for subsequent analysis. Second, beta mixture models were used to determine the methylation status of multiple genes. Last, to verify the existence of difference between the PCA samples and the corresponding non-tumor samples, the Wilcoxon rank-sum test was used as the measurement standard. Finally, mixture models and regression analyses of the DMGs were visualized, respectively.

### Functional and Pathway Enrichment Analysis of DMGs

Gene Ontology (GO), Kyoto Encyclopedia of Genes and Genomes (KEGG), and Gene Set Enrichment Analysis (GSEA) were used to explore the critical pathways associated with DMGs, which were performed using the R packages “org.Hs.eg.db” and “clusterProfiler.”

### Generation and Validation of the DMG-Based Classifier of BCR-Free Survival

To explore the relationship between the gene expression of DMGs and BCR-free survival, least absolute shrinkage and selector operation (LASSO) regression was performed to identify prognosis-related DMGs and establish a signature. Briefly, LASSO is a method that pushes regression coefficients toward zero *via* the application of an L1 penalty. If the penalty is larger, fewer predictors are selected, and as a result, several variables are diminished. In addition, analysis using the “glmnet” package based on the program with 1,000 iterations of Cox LASSO regression and 10-fold cross-validation led to seed genes being integrated into multiple gene sets. Seed genes with nonzero coefficients were identified as potential prognostic predictors. The linear combination of the regression coefficient (β) multiplied by its mRNA expression level can generate a risk score for candidate genes based on BCR (Tibshirani, 1997; Sauerbrei et al., 2007) as follows:

$$Riskscore=\overset{k}{\underset{i=1}{\sum }}\beta iSi$$

(k: the number of candidate genes, *β*i: the coefficient index of candidate genes, and Si: the expression level of candidate genes).

To classify patients into low-, medium-, and high-risk groups, the x-tile (Version 3.6.1) tool was used to determine the cut-off value of the risk score (Camp et al., 2004). Kaplan–Meier (K-M) survival plots and log-rank test were used to estimate BCR-free survival differences. To assess the effectiveness of the Classifier, the area under the curve (AUC) of the time-dependent receiver operating characteristic (ROC) curve was assessed. In this study, the predictive property was evaluated based on the time-dependent ROC curves, which were generated using the “survivalROC” and “rms” R package. In addition, the “ggplot2” R package was used for drawing.

### Screening of Prognostic Factors

To identify the meaningful predictive factors of a BCR-free state for PCA patients, univariate Cox regression analysis was performed with the Classifier (risk level) and clinicopathological features of patients. Additionally, multivariate Cox regression with 1000-times bootstrapping was performed using the “survival” package in R to eliminate confounding factors. The hazard ratio and its 95% CI for each variate were obtained. Statistical significance was set at a *p*-value < 0.05.

### Establishment and Validation of the Nomogram

The nomogram was constructed with meaningful predictive factors by multivariate Cox regression analysis. The calibration curves were plotted using the Hosmer–Lemeshow test, which was expected to calibrate the probability of patients with PCA after RP at 1, 3, and 5 years. Furthermore, the identification performance of the nomogram was quantified using Harrell’s concordance index (C-index). In total, 1,000 bootstrap resamples were processed for verification to obtain a stable C-index. The C-index ranged from 0.5 (indicative of poor or no predictive ability) to 1.0 (perfect predictive ability). A time-dependent ROC analysis (Heagerty et al., 2000) and area AUC were used to measure the predictive accuracy of the nomogram.

### External Validation of the Nomogram

The gene expression dataset (GSE21034), as a validation cohort, was downloaded from the GEO cohort (https://www.ncbi.nlm.nih.gov/geo/). The GSE21034 microarray dataset included gene expression profiles of 140 PCA samples and 29 nontumor samples as well as the related 140 clinicopathological features (Taylor et al., 2010) (GPL5188: Affymetrix Human Exon 1.0 ST Array). As previously mentioned, patients were classified into low-, medium-, and high-risk groups according to the cut-off value of the risk score determined using x-tile. K-M survival plots and log-rank test were used to evaluate the BCR-free survival differences. The time-dependent ROC analysis and AUC were used to measure the predictive accuracy of the nomogram, and the accuracy, sensitivity, and specificity of the model were quantitatively evaluated.

### Copy Number Variation, Mutation Features, and GSEA of the Candidate Genes

We collected graphic illustrations for CNVs and the seven-gene mutation profiles of all PCA tissues in the TCGA dataset, searching from the cBioPortal website (http://www.cbioportal.org/). Perl (strawberry-Perl-5.30.2.1) and GSEA 3.0 software (Gene sets database: c2. cp.kegg.v7.2. symbols.gmt) were used to perform GSEA analysis. Differences were considered statistically significant at an FDR <0.05.

### Cell Culture and DAC Treatment

The PCA cell line lymph node carcinoma of the prostate (LnCap) was purchased from Jining company (Shanghai, China) and maintained in minimum essential medium (cat no. C11875500BT; Gibco, Grand Island, NY, United States at 37°C and supplemented with 10% fetal bovine serum (cat no. A31608-02, Gibco) in a humidified atmosphere containing 5% CO_2_. LnCap cells in culture were treated with 5-aza-2ʹ-deoxycytidine (DAC, Cat No. A3656-5MG; Sigma-Aldrich, St. Louis, MO, United States) for 120 h, and the medium was replaced daily owing to DAC instability. For experiments involving DAC treatment, dimethyl sulfoxide was used as the control. The cells were harvested for extraction of genomic DNA and total RNA for analysis of DNAm and gene expression.

### RNA Extraction and Quantitative Reverse-Transcription PCR (qRT-PCR)

RNA extraction and qRT-PCR were performed using AG RNAex Pro Reagent (AG21101, Accurate Biology, Changsha, China). The samples were treated with 20% chloroform, vortexed briefly, and incubated at room temperature for 15 min. The samples were then centrifuged at high speed for 15 min at 4°C after the aqueous phase was transferred to a new tube, and an equal volume of isopropanol was added. Samples were incubated at room temperature for 10 min, followed by centrifugation at high speed for 10 min at 4°C. The pellets were then washed in 95% ethanol, dried, and resuspended in nuclease-free water. cDNA was synthesized using RNAiso plus reagent (Takara, Tokyo, Japan) according to the manufacturer’s instructions. qRT-PCR was performed using a LightCycler® 480 II (Roche, Basel, Switzerland) with a SYBR Green PCR kit (Takara Bio). The primer sequences are listed in Supplementary Table S6.

### Cancer Cell Line Encyclopedia Database

Gene expression of PCA cell lines was obtained from CCLE. We downloaded CCLE from the GEO dataset (Barretina et al., 2012). The gene expression profile GSE36133 (Affymetrix GPL15308 platform, Affymetrix Human Genome U133 plus 2.0 Array) was obtained. The probes were converted into the corresponding gene symbol according to the annotation information of the GPL571 platform. Genes with more than one probe set were averaged using R language.

### Statistical Analysis

The gene expression data of the seven DMGs were normalized using the TMM methods implemented in the package “edgeR.” The statistical analyses of qRT-PCR data were performed using R language (version 4.0.0) and GraphPad Prism 8.3.0. A *p*-value < 0.05 was considered statistically significant for two-sided tests.

## Results

### Identification of DEGs

**FIGURE 1:**
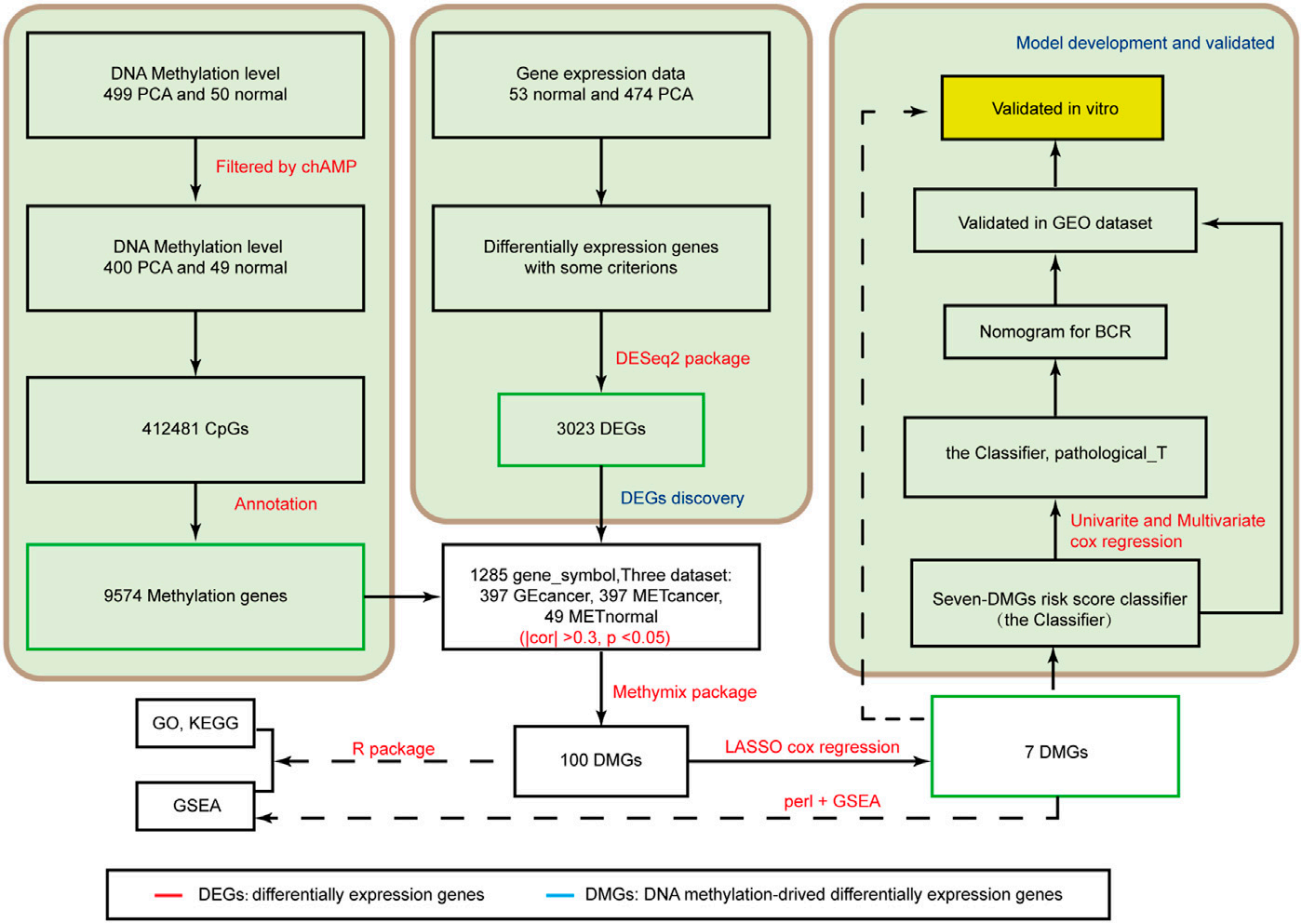
Analysis of the flowchart illustrates the exploration procedure for the PCA prognostic DMGs and establishment of risk score signature.

A flow diagram of the entire process is shown in Figure 1. By comparing the mRNA expression between PCA tissues and nontumorous prostate tissues, we identified 3,023 DEGs for further analysis. Among these DEGs, 1,262 were upregulated and 1761 were downregulated (Supplementary Table S2).

### Identification of DMGs

**FIGURE 2:**
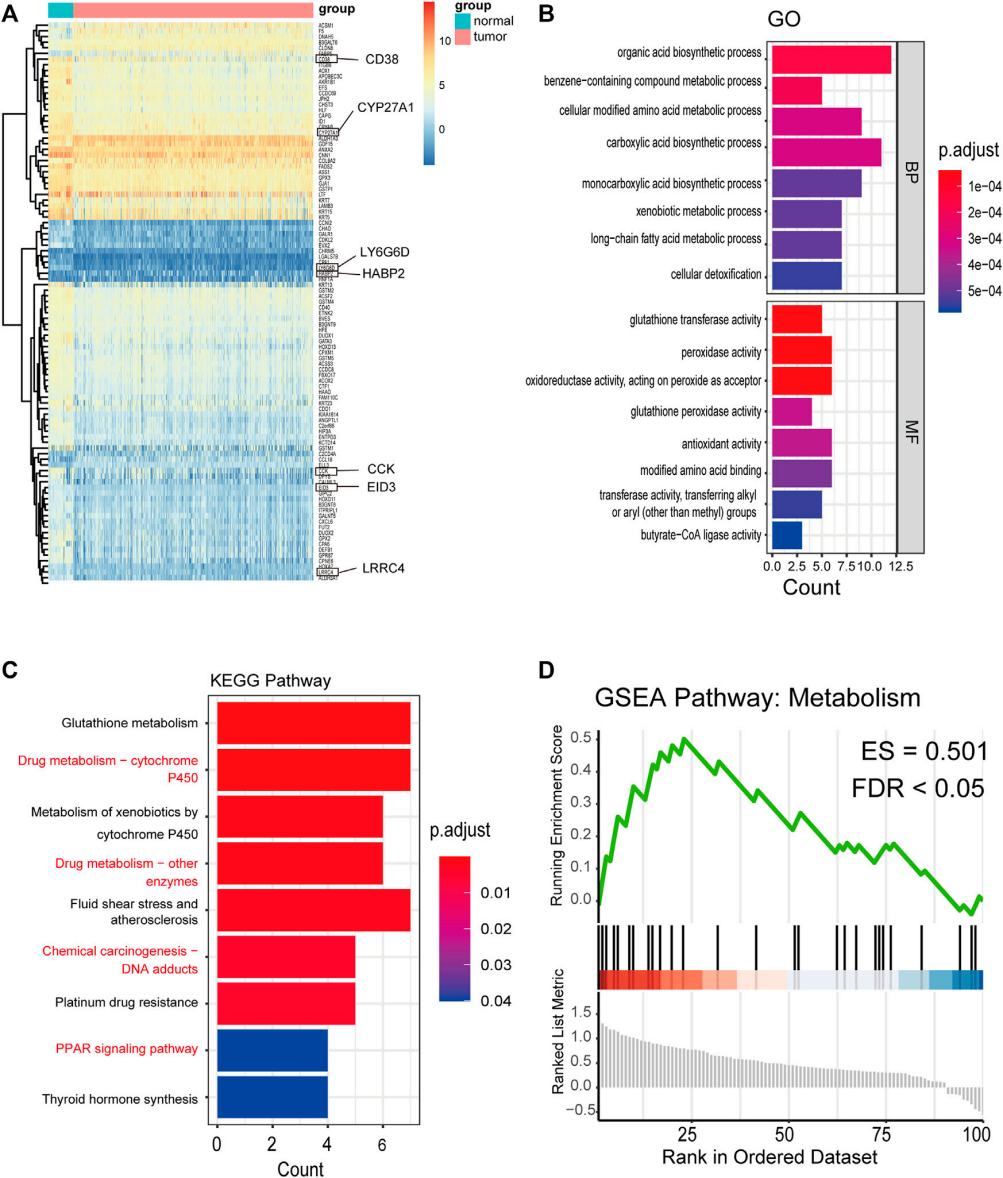
Distribution of the methylation level and gene expression of DNA methylation-driven genes and GO, KEGG, and GSEA pathways of 100 DMGs. **(A)** Distribution of the gene expression of 100 DMGs between PCA and nontumorous prostate tissues (seven representative genes are shown in the black frame). **(B)** GO analysis classified the DEGs into 2 groups (i.e., molecular function and biological process) and significant enriched GO Terms of 100 DMGs based on their functions. **(C)** KEGG pathway analysis. **(D)** GSEA KEGG pathway of 100 DMGs.

After identifying 9,574 methylated genes, we evaluated the level of methylation and gene expression level of 1,285 methylated genes from 397 PCA samples and the methylation level of these 1,285 methylation-associated genes from 49 non-tumor samples by integrating the datasets. The MethylMix (Gevaert, 2015) package was used to import these three datasets. Altogether, 100 DMGs were identified (Supplementary Table S3). Heatmap was used to show the gene expression (Figure 2A) of these 100 DMGs and took seven represented genes in black frames as an example. GO analyses were performed to elucidate the functional properties of the newly identified DMGs, and eight GO terms were obtained (Figure 2B), including the organic acid biosynthetic process, benzene-containing compound metabolic process, and cellular modified amino acid metabolic process (*p* < 0.001). Moreover, pathway analysis using the KEGG revealed that these genes were mainly enriched in glutathione metabolism, drug metabolism -cytochrome P450, platinum drug resistance, and the PPAR signaling pathway (*p* < 0.05; Figure 2C). KEGG pathway analysis revealed that the most abundant pathways were those related to metabolism and drug resistance. GSEA revealed that these genes were enriched in metabolism (Figure 2D).

### Establishment of a Classifier Related to BCR-Free Survival

**FIGURE 3:**
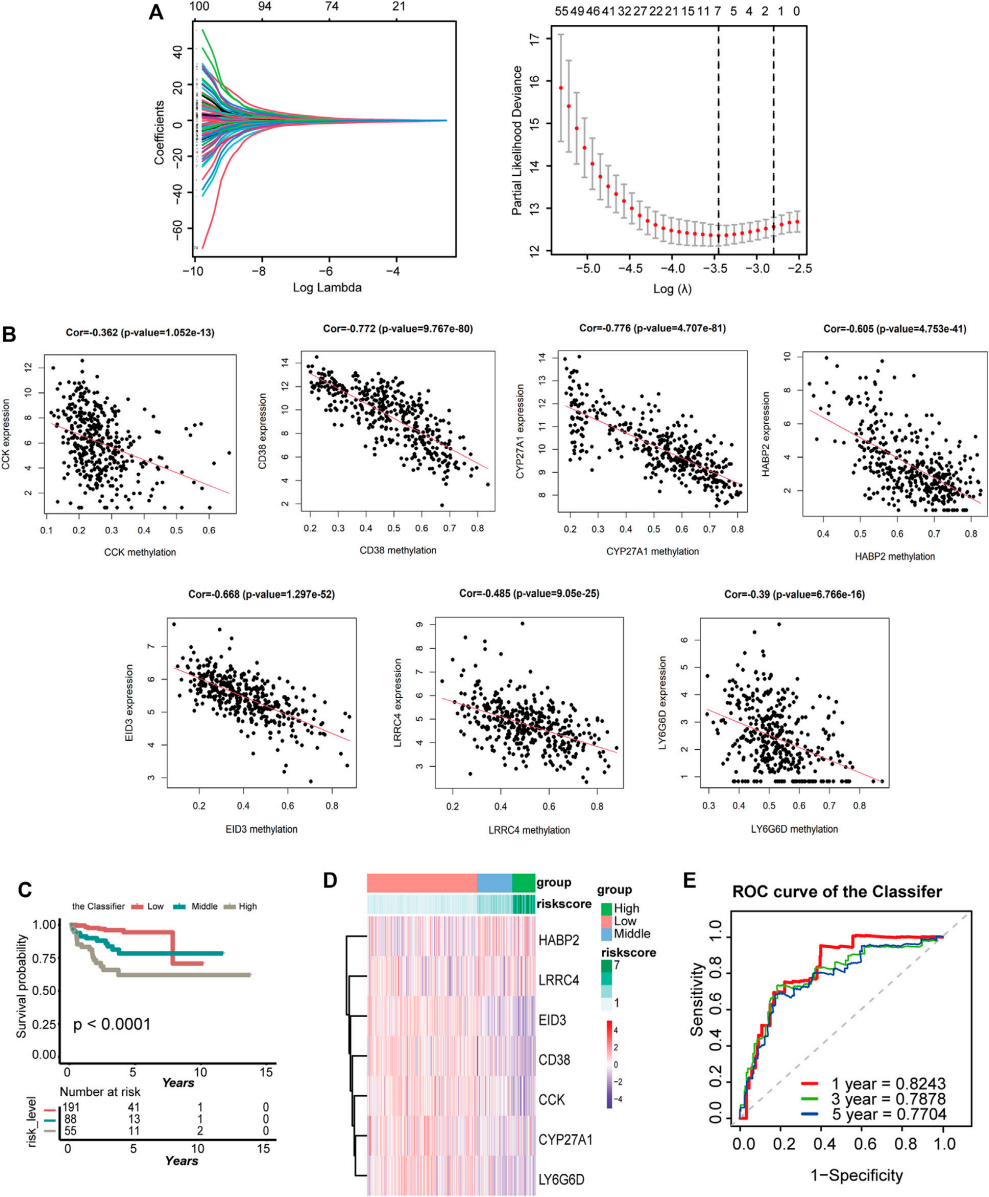
Establishment of the classifier based on seven DMGs in the TCGA cohort. **(A)** LASSO coefficient profiles of the 100 genes in TCGA cohort. A coefficient profile plot was generated against the log(lambda) sequence. Selection of the optimal parameter (lambda) in the LASSO model for TCGA-PRAD. A vertical line is drawn at the optimal value by 1−SE standards and results in seven nonzero coefficients. **(B)** Regression analysis between gene expression and DNA methylation of seven DMGs. **(C)** K-M survival curves compare BCR status among the low-, medium-, and high-expression groups of seven DMGs. **(D)** Heatmap of the seven DMGs expression profiles based on low-, medium-, and high-risk groups. **(E)** Time-dependent ROC for accuracy of BCR-free survival prediction by the seven-DMG signature (the Classifier) among 1, 3, and 5 years in TCGA group.

These 100 DMGs with 339 PCA samples with BCR time and status were included in LASSO analysis. Of these, *CCK, CD38, CYP27A1, EID3, HABP2, LRRC4,* and *LY6G6D* were recommended as candidate genes (Figure 3A). The methylation status of these seven genes was negatively correlated with gene expression (Figure3B). Among them, *CCK, CD38, CYP27A1, EID3, LRRC4,* and *LY6G6D* were hypermethylated, whereas *HABP2* was hypomethylated (Figure 3C, Supplementary Figure S3). Based on the seven genes, a formula for calculating the risk score was generated as follows:

Risk score = −0.066 × CCK mRNA level + (−0.127) × CD38 mRNA level + (−0.0615) × CYP27A1 mRNA level + (−0.833) × EID3 mRNA level + 0.088 × HABP2 mRNA level + 0.473 × LRRC4 mRNA level + (−0.122) × LY6G6D mRNA level.

Please note that the gene expression should be normalized before importing the formula (Supplementary File S1).

The range of the risk scores among the 334 patients in the TCGA dataset was between −4.588 and −1.81 (Supplementary Table S4). However, by analyzing the risk scores of the Classifier and BCR status, the patients with PCA could be classified into low-, medium-, and high-risk groups with the cut-off value from x-tile. In total, 191 patients with a cut-off value greater than −2.89 were included in the high-risk group, 88 patients with values between −3.33 and −2.89 were included in the medium-risk group, and 55 others were included in the low-risk group. K-M analyses of these three groups demonstrated that patients with lower risk scores had a lesser occurrence of BCR than those with medium-risk scores, which in turn had an even lower occurrence than those with high-risk scores (*p* < 0.0001; Figure 3D). The heatmap in Figure 3E shows the gene expression of the seven candidate genes based on the risk level. A time-dependent ROC curve was generated to describe the predictive ability of the Classifier, and the AUC values of the Classifier at 1, 3, and 5 years were 0.8243, 0.7878, and 0.7704, respectively (Figure 3F). As here, the same data were used to select genes and build the risk score, an association with BCR and the resulting predictive ability were to be expected.

### Establishment and Evaluation of the Nomogram for BCR-Free Survival Prediction in PCA

**FIGURE 4:**
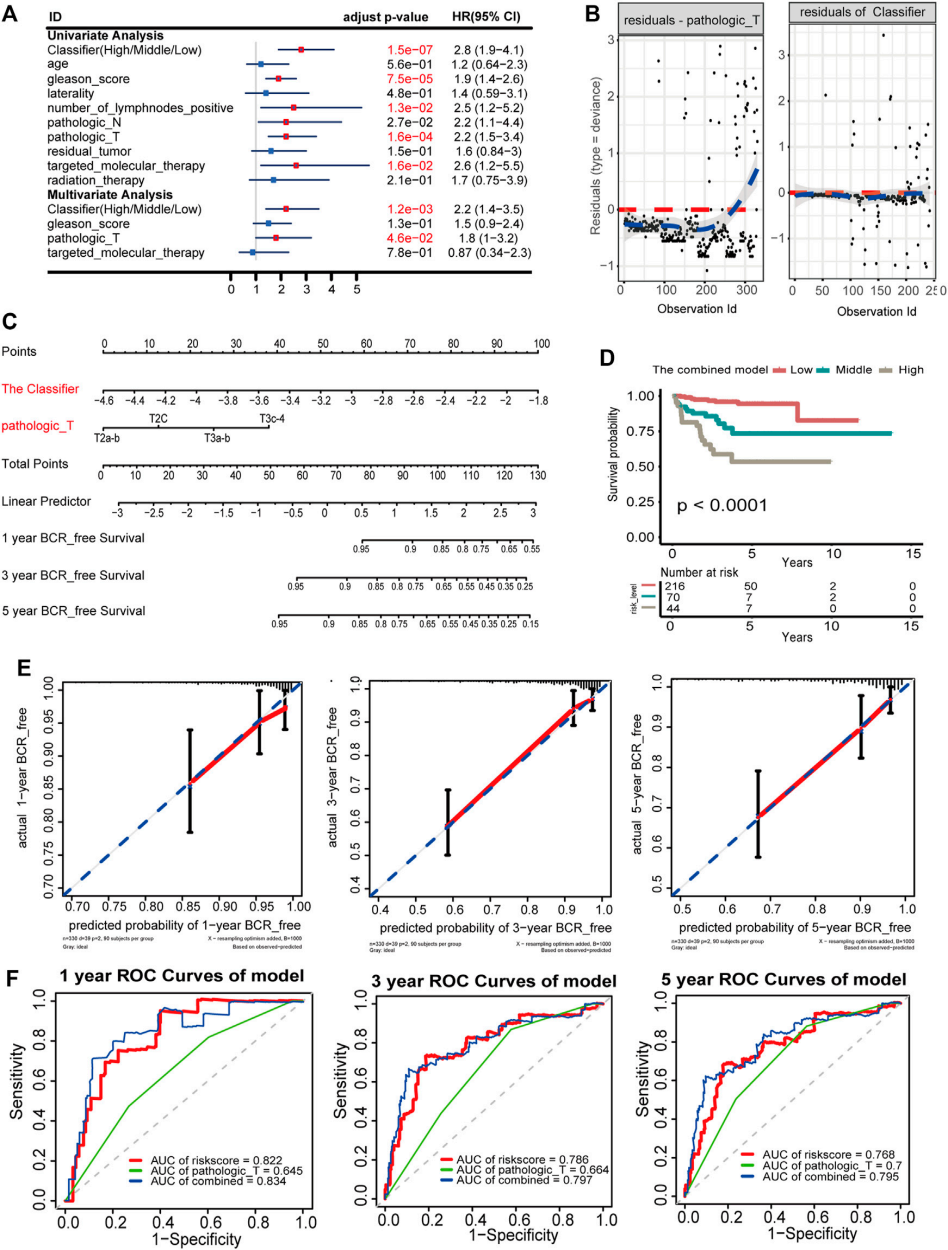
Nomogram to predict 1-, 3-, and 5-year BCR-free survival. The BCR-free survival nomogram was established in the TCGA cohort, incorporating pathological_T and the Classifier. **(A)** Univariate and multivariate analyses of the Classifier, clinical factors, and pathological characteristics with BCR-free survival. The statistical significance is indicated using different colors; red indicates statistical significance, and blue indicates no significance. **(B)** Schoenfeld residual suggested that this model met the equally proportional risk hypothesis. Schoenfeld model residuals versus pathological_T stage and the Classifier were plotted to obtain a preliminary assessment of whether these predictive factors should be incorporated into the model. **(C)** Nomogram to predict the 1-, 3-, and 5-year BCR-free survival of PCA patients. **(D)** K-M survival curves for comparison of BCR-free survival among the low-, medium-, and high-risk groups based on the combined model in the TCGA cohort. **(E)** Calibration curves of 1-, 3-, and 5-year BCR-free survival in the combined model. Blue dotted lines represent the ideal predictive model, and the solid red line represents the observed model. **(F)** Time-dependent ROC for accuracy of BCR-free survival prediction by the combined model among 1, 3, and 5 years.

The prognostic classifier (Classifier) and pathological_T were regarded as the key prognostic predictors using univariate and multivariate regression analyses (Figure 4A). Furthermore, the relationship between Schoenfeld model residuals and the Classifier was plotted to evaluate the importance of these prediction factors in the combined model. Schoenfeld residuals showed that the combined model satisfied the risk assumption of an equal proportion (Figure 4B). A nomogram was established based on the Classifier and pathological_T (Figure 4C). Based on the combined model, patients were divided into low-, medium-, and high-risk groups with the risk score from x-tile as the cut-off value (1.21 and 4.09). Patients with the lowest risk scores had the lowest BCR rates and those with the highest risk scores had the highest BCR rates when the K-M survival analysis was applied (*p* < 0.0001; Figure 4D). The C index and robust C- index values were 0.802 and 0.810, respectively, which means that the predicted results of the model were nearly consistent with the actual observed results. The calibration curve of the combined model for predicting BCR-free survival at 1, 3, and 5 years revealed favorable forecasting performance (Figure 4E). Additionally, the time-dependent ROC curve demonstrated that the AUC of the seven-DMG signature combined with the Classifier and pathological_T was significantly higher than that of the Classifier or Gleason score only at 1, 3, and 5 years (Figure 4F), indicating that the sensitivity of the nomogram was considerably better than that of the Classifier or the Gleason score alone. The nomogram offered excellent performance in BCR-free survival predictions, especially with a long term. Taken together, the findings suggest that the nomogram can help physicians provide appropriate recommendations for clinical therapy and follow-up schedules for patients with PCA.

### External Validation of the Nomogram

**FIGURE 5:**
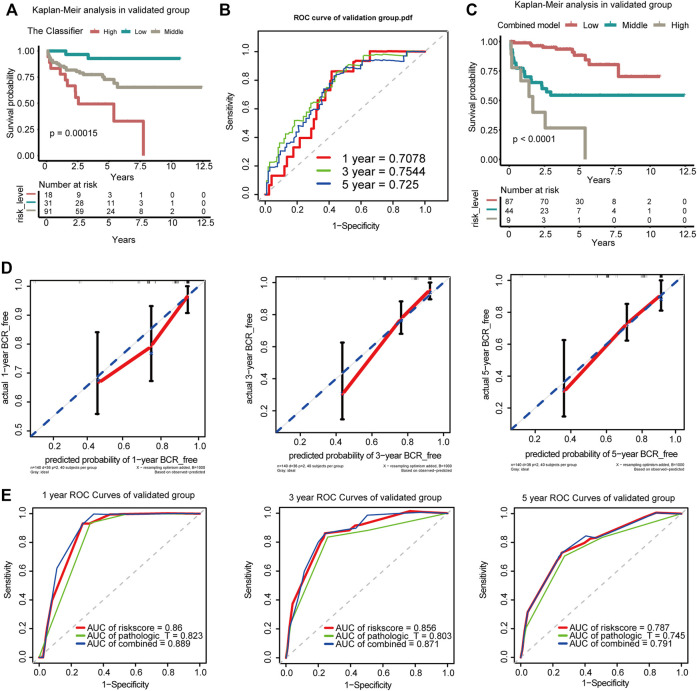
Verification of the Classifier and the combined model in the GEO dataset. **(A)** K-M survival curves for comparison of BCR-free survival among the low-risk, medium-risk, and high-risk score based on the Classifier. **(B)** Time-dependent ROC for accuracy of BCR-free survival prediction by the seven-DMG signature among 1, 3, and 5 years in the validated group. **(C)** K-M survival curves for comparison of BCR-free survival among the low-risk, medium-risk, and high-risk score based on the combined model. **(D)** Calibration curves of 1-, 3-, and 5-year BCR-free survival. Blue dotted lines represent the ideal predictive model, and the solid red line represents the observed model. **(E)** Time-dependent ROC analysis was used to evaluate the accuracy of the BCR-free survival nomograms. The red, blue, and green solid lines represent the combined model, GS, and Classifier, respectively.

The GEO dataset GSE21034 was subsequently used to verify the newly established nomogram. In total, 140 cases were included in the external study (Supplementary Table S5). Based on the Classifier, patients were divided into low-, medium-, and high-risk groups with the risk score from x-tile as the cut-off value (0.02 and 0.03). Generally, comparing the three cohorts, patients with the lowest risk scores had lowest BCR rates and those with the highest risk scores had the highest BCR rates when the K-M survival analysis was applied (*p* = 0.00015; Figure 5A). The AUCs of BCR-free survival at 1-, 3-, and 5-year BCR-free survival were 0.7078, 0.7544, and 0.725, respectively (Figure 5B). Based on the combined model of the Classifier and pathologic_T, patients were divided into low-, medium-, and high-risk groups with the risk score from x-tile as the cut-off value (0.52 and 2.12). Comparing the three cohorts, patients with the lowest risk scores had the lowest BCR rates and those with the highest risk score had the highest BCR rates when the K-M survival analysis was applied. (*p* < 0.0001; Figure 5C).

The calibration curves for 1-, 3-, and 5-year BCR-free survival status based on the nomogram suggested a significant agreement between the predicted outcomes and those observed in the validation group (Figure 5D). The combined model of the Classifier and pathological_T exhibited better predictive ability than either the Classifier or pathological_T alone. The AUCs of 1-, 3-, and 5-year BCR-free survival were 0.889, 0.871, and 0.791, respectively, in our validation group (Figure 5E). The 1-, 3-, and 5-year trend in the AUC in the validation cohort was consistent with that in the TCGA cohort, which further illustrates the value of the prediction model for long-term follow-up. Additionally, coincidence analysis of the combined model showed that the C-index was 0.853 and the robust C-index was 0.860.

### CNV, Mutation Features, and KEGG Signaling Pathway Based on GSEA

**FIGURE 6:**
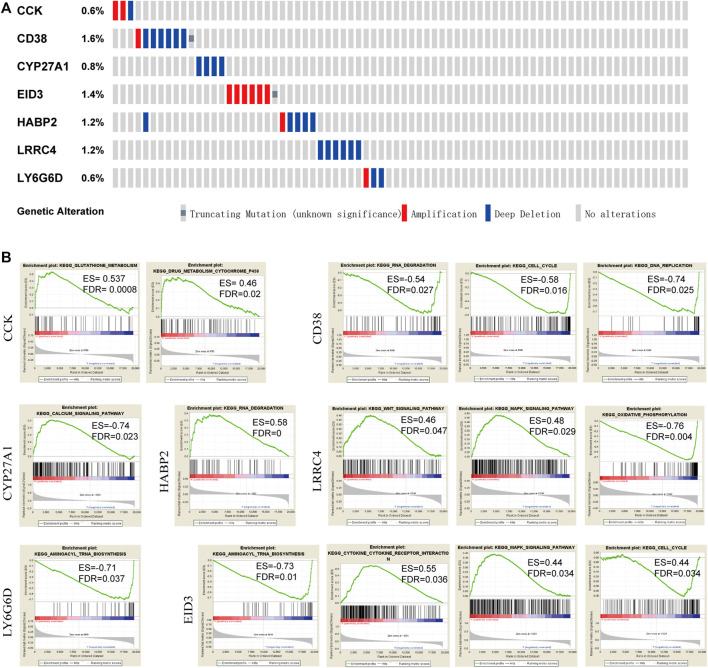
Genetic changes and mutation features of seven DMGs by cBioPortal and pathways by GSEA. **(A)** Genetic alterations of DMGs in PCA samples. Rows and columns represent the genes and tumor samples, respectively. **(B)** KEGG pathways enrichment analysis in each of the seven genes based on GSEA (FDR <0.05).

The seven candidate DMGs were affected by methylation, gene amplification, deletion, and mutation. We noted that the rates of genetic alterations among these seven genes were between 0.8 and 1.6% based on the GDC TCGA-PRAD database (Figure 6A), indicating that the effect of methylation might promote a change in gene expression.

To determine the potential signaling pathways affecting these seven genes, functional category enrichment analysis was performed to examine their function. *CCK* was mainly related to glutathione metabolism, drug metabolism, and cytochrome P450. *CD38* was mainly associated with RNA degradation, the cell cycle, and DNA replication. *CYP27A1* was mainly associated with the calcium signaling pathway. *HABP2* was mainly associated with RNA degradation. *LRRC4* was mainly associated with the WNT signaling pathway, the MAPK signaling pathway, and oxidative phosphorylation, and *LY6G6D* was mainly associated with aminoacyl tRNA biosynthesis. *EID3* was mainly associated with aminoacyl tRNA biosynthesis, cytokine receptor interaction, the MAPK signaling pathway, and the cell cycle. A NOM q-value (FDR) < 0.05 was set as the threshold value (Figure 6B).

### Expression of Seven DMGs in DAC-Treated LnCap Cells

**FIGURE 7:**
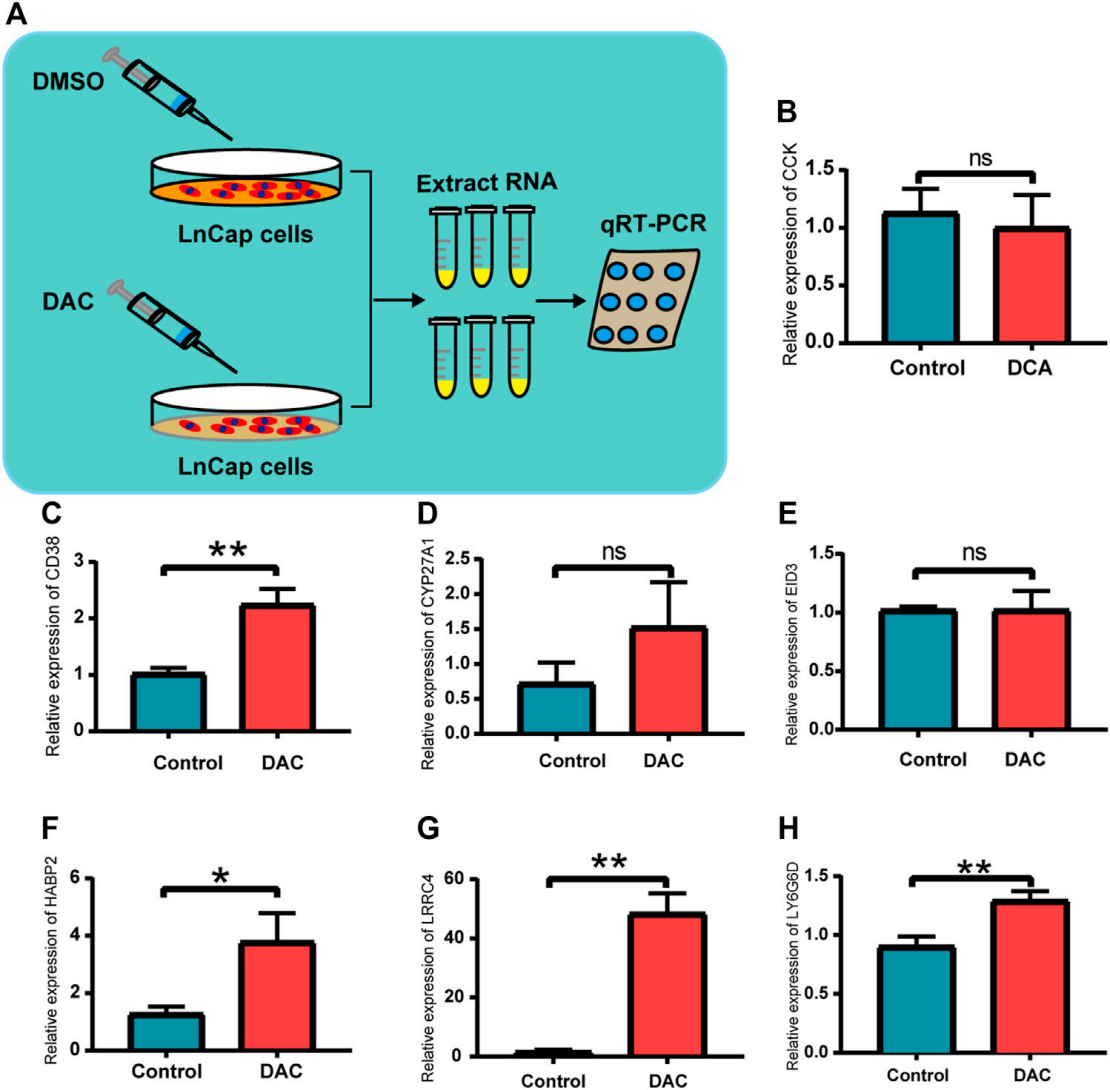
Validation in prostate cancer cells for the seven DMGs. **(A)** Schematic illustration of demethylation of LnCap after DAC treatment. **(B–H)** Relative expression of CCK, CD38, CYP27A1, EID3, HABP2, LRRC4, and LY6G6D between the DAC group and the control; ns: *p* > 0.05, *p < 0.05, **p < 0.01.

As shown in Figure 3B and Supplementary Figure S3, the methylation levels of *CCK, CD38, CYP27A1, EID3, HABP2, LRRC4,* and *LY6G6D* exhibited the strongest negative correlation with their gene expression, respectively. To confirm this, we analyzed the changes in the expression of the four genes in DAC-treated LNCaP cells to evaluate their functional correlation with methylation (Figure 7A). Our results indicated that the expression of *CD38, HABP2, LRRC4,* and *LY6G6D* was upregulated in LnCap cells treated with DAC, whereas that of *CCK, CYP27A1,* and *EID3* did not significantly change (Figures 7B–H). The results were thus not entirely validated using the LNCaP cells’ data.

## Discussion

The course of PCA is long after operation, and patients often want to know when the next recurrence will occur so that they can be treated for it as soon as possible (Bianco et al., 2005). Predicting the BCR of PCA is clinically essential, but it is difficult for currently available prediction tools to meet current clinical needs. Thus, considerable effort has been devoted to exploring new technologies to detect early signs of tumors (Wu and Qu, 2015). This study attempted to predict BCR from a new perspective of epigenetic DMGs. We successfully established a predictive model based on seven DMGs to determine low-, medium-, and high-risk groups from the TCGA and GEO datasets. In addition, based on the Classifier, a nomogram was constructed to predict the BCR-free survival rate, which almost unambiguously classified patients into low-, medium-, and high-risk groups and distinguished BCR-free survival with high accuracy, achieving high sensitivity and specificity. Taken together, the findings suggest that the nomogram has the potential to predict BCR in patients with PCA after RP.

Brockman et al. (2015) constructed a nomogram that showed excellent predictive value for BCR, but this nomogram was built based on PSA, which limits its sensitivity at low PSA levels (Fendler et al., 2019). ^99m^Tc-MIP-1404 PSMA-SPECT/CT was also shown to have high performance for detecting PSMA-positive lesions suggestive of tumor recurrence in patients with PCA BCR and very low serum PSA levels (Schmidkonz et al., 2019). However, it is an invasive examination, which limits its practical application. In contrast, our prediction tool is based mainly on the pathological_T and the Classifier. Most of the specimens collected were postoperative specimens that were not affected by PSA. Thus, the nomogram can use postoperative specimens to detect DMGs to avoid postoperative invasive puncture and unpredictability with low PSA levels.

Regarding the seven DMGs, cholecystokinin, also named *CCK*, as a gastrointestinal hormone, is a chemical messenger that regulates the physiological functions of the intestine and pancreas, including secretion, motility, absorption, and digestion (Thomas et al., 2003). The cholecystokinin hormones affect proliferation by blocking their respective receptors in PCA (Thomas et al., 2003). Moreover, as early as 1997, Jean Claude Reubi studied the role of *CCK-A* and *CCK-B* in some neuroendocrine and reproductive tumors, including PCA (Reubi et al., 1997). In addition, we found that CCK is mainly related to glutathione metabolism and drug metabolism. This suggests that this might be the beginning of a new understanding of *CCK* in neuroendocrine PCA. *CD38* is a glycoprotein that regulates cellular nicotinamide adenine dinucleotide metabolism. One study suggested that the methylation of *CD38* regulates the progression of localized and metastatic PCA. In our study, *CD38* was mainly associated with RNA degradation, the cell cycle, and DNA replication. *CYP27A1* is an enzyme that stimulates the transformation of cholesterol to oxysterol 27-hydroxycholesterol (27-HC). Accumulating evidence suggests that 27-HC acts as an agonist of the estrogen receptor. Moreover, *CYP27A1* is associated with the risk of lethal PCA, another sex hormone–dependent tumor (Shui et al., 2012). The relationship between *CYP27A1* methylation and PCA has not been reported to date. Interestingly, one study showed that the excessive corticosterone-induced downregulation of *CYP27A1* coincides significantly with increased CpG methylation of its promoters (Hu et al., 2017). In this study, *CYP27A1* methylation was associated with the calcium signaling pathway. De-regulation of calcium signals in prostate tumor cells mediates several pathological dysfunctions associated with PCA progression, which plays a relevant role in tumor cell death, proliferation, motility invasion, and tumor metastasis (Ardura et al., 2020).

Furthermore, *HABP2, LRRC4, LY6G6D,* and *EID3* had not been studied in PCA to date. *HABP2* has mostly been studied in thyroid cancer (Zhao et al., 2015; Zhang and Xing, 2016). However, it is expected to be studied in PCA, another endocrine-dependent cancer. As a tumor suppressor gene, inactivation of LRRC4 mediates DNA hypermethylation in central nervous system tumors (Zhang et al., 2008). In our study, *LRRC4* was mainly associated with the WNT signaling pathway, the MAPK signaling pathway, and oxidative phosphorylation, which are the common pathways in the progression of PCA (Schöpf et al., 2016; Murillo-Garzón and Kypta, 2017; Park et al., 2020). *LY6G6D* can lead to the progression of colorectal cancer and colon adenocarcinoma (Sewda et al., 2016; Giordano et al., 2019). However, its relationship with PCA needs further study. High expression of EID3 is an adverse prognostic indicator for patients with colorectal cancer (Munakata et al., 2016). In our study, EID3 was mainly associated with aminoacyl tRNA biosynthesis, cytokine–cytokine receptor interaction, the MAPK signaling pathway, and the cell cycle, which requires further study for validation. In addition, we further studied these seven genes as DMGs in LnCap cells. The changes in the expression of *CD38, HABP2, LRRC4,* and *LY6G6D* after DAC demethylation were statistically significant, whereas *CCK, CYP27A1,* and *EID3* were not statistically significant. We searched the datasets of GSE36,133 and GSE21034 and found that the gene expression of the seven DMGs was also found in other prostate cancer cell lines (Supplementary Table S7, Supplementary Table S8). Thus, other cell lines might be included for validation in the future.

Notably, this new nomogram excluded the Gleason score, N staging, and the number of positive lymph nodes from being considered as the predictive factors. However, the pathological stage markedly contributes to the predisposition of distant metastasis (Pound et al., 1999). T staging, Gleason score, and PSA can be evaluated to precisely predict the BCR risk stratification of PCA (Eisenberg et al., 2010). Although T staging was included, it had limited impact on the model according to the AUC. The number of lymph node metastases also significantly affected the survival time of patients with PCA. However, Felix Preisser et al. (2020) suggested that there was no significant difference in clinical outcomes in patients with D'Amico high- or intermediate-risk PCA who had or had not undergone pelvic lymph node dissection during radical prostatectomy. Therefore, the therapeutic benefits of pelvic lymph node dissection remain elusive (Preisser et al., 2020). This observation corroborates our finding from the nomogram on early PCA, as it also excludes the influence of the positive lymph nodes. In addition, a positive surgical margin was also an effective predictor of BCR ≥5 years post-surgery (Negishi et al., 2017). However, our prediction model did not take this into account. This may be due to the weakening of the function of this project after the replacement of DNAm biomarkers.

A limitation of this study is that the data obtained were from TCGA and GEO datasets only, and it lacks further validation using third-party clinical data. In addition, family history and ethnic/ethnic background are closely associated with PCA morbidity and affect it significantly. Hence, further investigations are warranted to conclusively establish whether the nomogram is applicable to the Asian population or not. This can be addressed by verifying the function of the Classifier with relevant data. Furthermore, owing to the lack of *in vivo* validation of our data, further evaluation of altered expression of these genes in cancer tissues compared to the normal tissues is required.

## Conclusion

In this study, we constructed a nomogram based on DMGs that can predict postoperative BCR of PCA with high sensitivity and specificity, which expands our understanding of DMGs in the pathogenesis of PCA. The target genes had high clinical specificity and may function as a molecular marker and a potential therapeutic target for PCA in the future. However, these results were not validated using the data obtained from the LNCaP cells. Further verification using clinical and experimental data is required.

## Data Availability

The original contributions presented in the study are included in the article/Supplementary Material; further inquiries can be directed to the corresponding authors.
