# Supplementary material for: Clinical and Biological Significance of DNA Methylation-Driven Differentially Expressed Genes in Biochemical Recurrence After Radical Prostatectomy
Source: Front Genet. 2022 Feb 2;13:727307. doi: 10.3389/fgene.2022.727307 (PMC8847683; doi:10.3389/fgene.2022.727307)
Supplement: Supplementary file 3 [file Table2.DOCX]

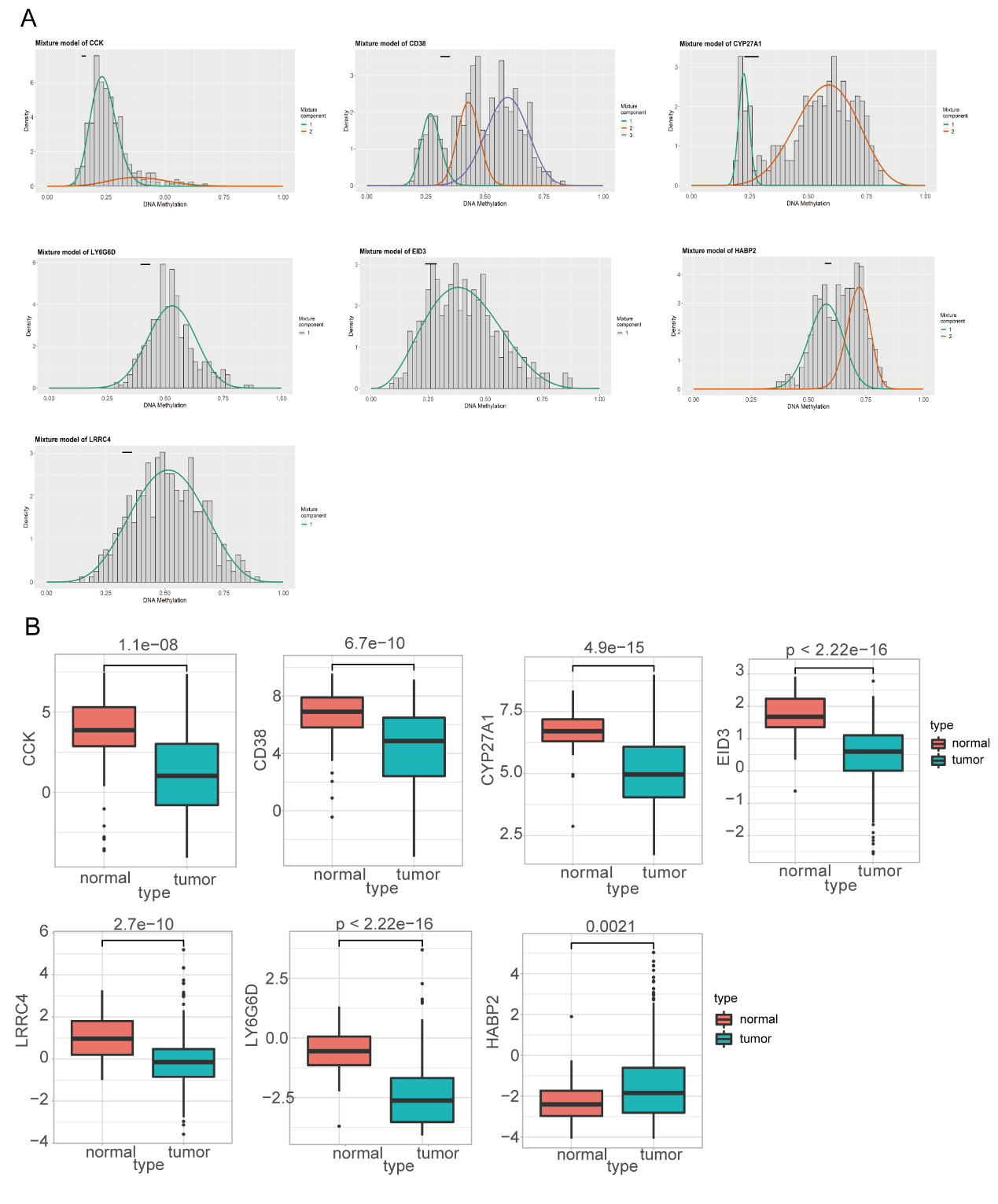


**Figure S3**. Methylation level and gene expression of seven DMGs between PCA and normal tissue. **(A)** Mixture models for seven genes. The horizontal black bar indicates the distribution of methylation values in normal samples. The histogram illustrates the distribution of methylation in tumor samples (signified as beta values, where higher beta values denote more significant methylation). **(B)** The gene expression of seven DMGs between the PCA and normal tissue.

**Table S1. The accession number of the sample set from TCGA.**

| The accession number of RNA-sequencing profiles of 474 PRAD samples | ID |  |
| --- | --- | --- |
|  | TCGA.G9.6496.01A | TCGA.HC.A4ZV.01A |
|  | TCGA.VP.A879.01A | TCGA.HI.7169.01A |
|  | TCGA.HC.7080.01A | TCGA.G9.7509.01A |
|  | TCGA.EJ.5501.01A | TCGA.KC.A7FE.01A |
|  | TCGA.HC.8213.01A | TCGA.XK.AAJA.01A |
|  | TCGA.HC.7744.01A | TCGA.G9.7521.01A |
|  | TCGA.XK.AAK1.01A | TCGA.EJ.A8FN.01A |
|  | TCGA.HC.7747.01A | TCGA.EJ.5499.01A |
|  | TCGA.ZG.A8QW.01A | TCGA.EJ.A46F.01A |
|  | TCGA.CH.5746.01A | TCGA.EJ.8470.01A |
|  | TCGA.G9.6384.01A | TCGA.FC.7961.01A |
|  | TCGA.G9.6367.01A | TCGA.YL.A8HK.01A |
|  | TCGA.YL.A8HO.01A | TCGA.HC.7748.01A |
|  | TCGA.EJ.A6RC.01A | TCGA.G9.7522.01A |
|  | TCGA.G9.6342.01A | TCGA.CH.5752.01A |
|  | TCGA.EJ.7331.01A | TCGA.KK.A7AP.01A |
|  | TCGA.EJ.7783.01A | TCGA.KK.A6E4.01A |
|  | TCGA.J4.A67S.01A | TCGA.HC.A632.01A |
|  | TCGA.V1.A9OL.01A | TCGA.EJ.7321.01A |
|  | TCGA.HC.7745.01A | TCGA.EJ.A8FU.01A |
|  | TCGA.MG.AAMC.01A | TCGA.EJ.5518.01A |
|  | TCGA.G9.6348.01A | TCGA.EJ.5524.01A |
|  | TCGA.G9.6338.01A | TCGA.J4.8198.01A |
|  | TCGA.EJ.7791.01A | TCGA.KK.A8IM.01A |
|  | TCGA.M7.A71Y.01A | TCGA.CH.5748.01A |
|  | TCGA.XQ.A8TB.01A | TCGA.VP.A87J.01A |
|  | TCGA.M7.A725.01A | TCGA.HC.7231.01A |
|  | TCGA.ZG.A9LU.01A | TCGA.2A.A8W1.01A |
|  | TCGA.KK.A6E7.01A | TCGA.J4.A67T.01A |
|  | TCGA.KK.A8IJ.01A | TCGA.CH.5739.01A |
|  | TCGA.2A.A8VV.01A | TCGA.ZG.A9L1.01A |
|  | TCGA.V1.A8MG.01A | TCGA.HC.A6AN.01A |
|  | TCGA.EJ.A65B.01A | TCGA.KK.A5A1.01A |
|  | TCGA.HC.8262.01A | TCGA.EJ.5504.01A |
|  | TCGA.J4.A83K.01A | TCGA.EJ.7314.01A |
|  | TCGA.KK.A8I8.01A | TCGA.QU.A6IM.01A |
|  | TCGA.Y6.A8TL.01A | TCGA.ZG.A8QZ.01A |
|  | TCGA.KK.A7AZ.01A | TCGA.HC.8261.01A |
|  | TCGA.HC.8256.01A | TCGA.EJ.8469.01A |
|  | TCGA.HC.7738.01A | TCGA.2A.A8W3.01A |
|  | TCGA.HC.8265.01A | TCGA.J9.A8CL.01A |
|  | TCGA.HI.7171.01A | TCGA.KK.A7AQ.01A |
|  | TCGA.CH.5794.01A | TCGA.EJ.A65D.01A |
|  | TCGA.HC.7233.01A | TCGA.EJ.5519.01A |
|  | TCGA.EJ.7115.01A | TCGA.YL.A8HM.01A |
|  | TCGA.EJ.A8FP.01A | TCGA.M7.A721.01A |
|  | TCGA.EJ.5505.01A | TCGA.V1.A8WL.01A |
|  | TCGA.CH.5738.01A | TCGA.KK.A7AV.01A |
|  | TCGA.QU.A6IO.01A | TCGA.G9.6370.01A |
|  | TCGA.HC.7818.01A | TCGA.EJ.5514.01A |
|  | TCGA.CH.5771.01A | TCGA.VP.A87K.01A |
|  | TCGA.HC.A6AS.01A | TCGA.ZG.A9MC.01A |
|  | TCGA.XK.AAJ3.01A | TCGA.EJ.AB20.01A |
|  | TCGA.EJ.5512.01A | TCGA.XK.AAIR.01A |
|  | TCGA.H9.A6BY.01A | TCGA.V1.A8ML.01A |
|  | TCGA.KC.A4BV.01A | TCGA.KK.A8IB.01A |
|  | TCGA.G9.6369.01A | TCGA.J4.A67N.01A |
|  | TCGA.EJ.A7NM.01A | TCGA.EJ.5516.01A |
|  | TCGA.KK.A59Y.01A | TCGA.V1.A9OF.01A |
|  | TCGA.KK.A8IK.01A | TCGA.YL.A8SB.01A |
|  | TCGA.KK.A7B0.01A | TCGA.EJ.A7NN.01A |
|  | TCGA.G9.A9S0.01A | TCGA.HC.7212.01A |
|  | TCGA.CH.5769.01A | TCGA.V1.A9O5.01A |
|  | TCGA.G9.6366.01A | TCGA.HC.A8D0.01A |
|  | TCGA.EJ.7786.01A | TCGA.2A.A8VT.01A |
|  | TCGA.VP.A875.01A | TCGA.ZG.A9LB.01A |
|  | TCGA.EJ.A46D.01A | TCGA.HC.7079.01A |
|  | TCGA.J4.A67L.01A | TCGA.HC.A6AP.01A |
|  | TCGA.G9.6363.01A | TCGA.KC.A4BR.01A |
|  | TCGA.HC.A9TE.01A | TCGA.V1.A9OA.01A |
|  | TCGA.J4.A6G3.01A | TCGA.EJ.A46H.01A |
|  | TCGA.KK.A7B3.01A | TCGA.EJ.5507.01A |
|  | TCGA.YL.A8HJ.01A | TCGA.KK.A8ID.01A |
|  | TCGA.ZG.A9NI.01A | TCGA.XK.AAJR.01A |
|  | TCGA.VN.A88R.01A | TCGA.HC.7750.01A |
|  | TCGA.EJ.5530.01A | TCGA.G9.6347.01A |
|  | TCGA.HC.7752.01A | TCGA.EJ.5522.01A |
|  | TCGA.EJ.5521.01A | TCGA.HC.A631.01A |
|  | TCGA.YL.A9WX.01A | TCGA.CH.5763.01A |
|  | TCGA.KK.A6E5.01A | TCGA.EJ.7794.01A |
|  | TCGA.TP.A8TT.01A | TCGA.V1.A9Z8.01A |
|  | TCGA.KK.A7AY.01A | TCGA.EJ.7788.01A |
|  | TCGA.HC.7213.01A | TCGA.EJ.5495.01A |
|  | TCGA.EJ.5517.01A | TCGA.V1.A8MU.01A |
|  | TCGA.CH.5751.01A | TCGA.KK.A59X.01A |
|  | TCGA.HC.7820.01A | TCGA.G9.6498.01A |
|  | TCGA.J4.A6G1.01A | TCGA.G9.6378.01A |
|  | TCGA.EJ.5503.01A | TCGA.G9.7523.01A |
|  | TCGA.YL.A8SA.01A | TCGA.G9.6364.01A |
|  | TCGA.TP.A8TV.01A | TCGA.KK.A8IH.01A |
|  | TCGA.ZG.A9LM.01A | TCGA.VP.A87B.01A |
|  | TCGA.J4.A6M7.01A | TCGA.EJ.5494.01A |
|  | TCGA.ZG.A9M4.01A | TCGA.EJ.5502.01A |
|  | TCGA.CH.5745.01A | TCGA.KC.A7FD.01A |
|  | TCGA.KK.A8IG.01A | TCGA.VN.A943.01A |
|  | TCGA.HC.7821.01A | TCGA.KK.A6E3.01A |
|  | TCGA.FC.A4JI.01A | TCGA.KK.A7AW.01A |
|  | TCGA.G9.6354.01A | TCGA.HC.8216.01A |
|  | TCGA.EJ.A7NK.01A | TCGA.M7.A722.01A |
|  | TCGA.KK.A8I5.01A | TCGA.CH.5740.01A |
|  | TCGA.VP.A876.01A | TCGA.J9.A8CK.01A |
|  | TCGA.YJ.A8SW.01A | TCGA.EJ.7792.01A |
|  | TCGA.G9.6365.01A | TCGA.G9.6332.01A |
|  | TCGA.2A.A8VO.01A | TCGA.EJ.7784.01A |
|  | TCGA.EJ.7789.01A | TCGA.J4.AATZ.01A |
|  | TCGA.V1.A8WN.01A | TCGA.J4.A83M.01A |
|  | TCGA.G9.6343.01A | TCGA.J9.A52E.01A |
|  | TCGA.EJ.A8FO.01A | TCGA.CH.5762.01A |
|  | TCGA.HI.7168.01A | TCGA.G9.A9S7.01A |
|  | TCGA.EJ.7785.01A | TCGA.J4.AAU2.01A |
|  | TCGA.J4.A67M.01A | TCGA.KC.A7FA.01A |
|  | TCGA.CH.5753.01A | TCGA.HC.7736.01A |
|  | TCGA.KC.A7F5.01A | TCGA.ZG.A9ND.01A |
|  | TCGA.HC.A9TH.01A | TCGA.VN.A88O.01A |
|  | TCGA.HC.A6AL.01A | TCGA.G9.6494.01A |
|  | TCGA.2A.AAYU.01A | TCGA.EJ.5515.01A |
|  | TCGA.YL.A8S8.01A | TCGA.V1.A8WW.01A |
|  | TCGA.KK.A6E1.01A | TCGA.KK.A7B2.01A |
|  | TCGA.TK.A8OK.01A | TCGA.V1.A9OQ.01A |
|  | TCGA.EJ.8472.01A | TCGA.V1.A8MK.01A |
|  | TCGA.EJ.7797.01A | TCGA.EJ.7793.01A |
|  | TCGA.HC.7230.01A | TCGA.G9.7510.01A |
|  | TCGA.XJ.A9DQ.01A | TCGA.J4.A67R.01A |
|  | TCGA.J9.A52B.01A | TCGA.J4.A83L.01A |
|  | TCGA.EJ.5511.01A | TCGA.H9.A6BX.01A |
|  | TCGA.EJ.5527.01A | TCGA.CH.5790.01A |
|  | TCGA.YL.A8S9.01A | TCGA.HC.8259.01A |
|  | TCGA.J4.A67O.01A | TCGA.ZG.A9L6.01A |
|  | TCGA.V1.A9O9.01A | TCGA.VN.A88Q.01A |
|  | TCGA.CH.5791.01A | TCGA.KC.A4BN.01A |
|  | TCGA.V1.A8MM.01A | TCGA.J9.A52D.01A |
|  | TCGA.FC.7708.01A | TCGA.HC.7209.01A |
|  | TCGA.ZG.A8QY.01A | TCGA.CH.5750.01A |
|  | TCGA.EJ.7328.01A | TCGA.G9.6356.01A |
|  | TCGA.EJ.5510.01A | TCGA.ZG.A9KY.01A |
|  | TCGA.EJ.5498.01A | TCGA.HC.7078.01A |
|  | TCGA.VN.A88P.01A | TCGA.V1.A9ZI.01A |
|  | TCGA.EJ.A65J.01A | TCGA.HC.7211.01A |
|  | TCGA.EJ.8474.01A | TCGA.HC.7232.01A |
|  | TCGA.KK.A8IF.01A | TCGA.J4.A83J.01A |
|  | TCGA.G9.6339.01A | TCGA.HC.7737.01A |
|  | TCGA.HC.7081.01A | TCGA.ZG.A9LY.01A |
|  | TCGA.XJ.A9DX.01A | TCGA.EJ.A7NF.01A |
|  | TCGA.KK.A7B4.01A | TCGA.CH.5765.01A |
|  | TCGA.V1.A8X3.01A | TCGA.EJ.A7NG.01A |
|  | TCGA.J4.A83N.01A | TCGA.ZG.A9LN.01A |
|  | TCGA.XA.A8JR.01A | TCGA.HC.8260.01A |
|  | TCGA.EJ.7315.01A | TCGA.XJ.A83G.01A |
|  | TCGA.M7.A724.01A | TCGA.KK.A8IL.01A |
|  | TCGA.EJ.A65M.01A | TCGA.J9.A8CM.01A |
|  | TCGA.G9.7519.01A | TCGA.EJ.7781.01A |
|  | TCGA.EJ.A7NH.01A | TCGA.V1.A9OH.01A |
|  | TCGA.J4.A83I.01A | TCGA.WW.A8ZI.01A |
|  | TCGA.EJ.A6RA.01A | TCGA.XK.AAJP.01A |
|  | TCGA.HC.8257.01A | TCGA.CH.5754.01A |
|  | TCGA.VP.A87C.01A | TCGA.CH.5766.01A |
|  | TCGA.CH.5744.01A | TCGA.CH.5792.01A |
|  | TCGA.V1.A8WV.01A | TCGA.CH.5743.01A |
|  | TCGA.VP.A872.01A | TCGA.ZG.A9L4.01A |
|  | TCGA.J4.AATV.01A | TCGA.H9.7775.01A |
|  | TCGA.V1.A8MF.01A | TCGA.J4.8200.01A |
|  | TCGA.V1.A9Z9.01A | TCGA.EJ.5497.01A |
|  | TCGA.KK.A8I6.01A | TCGA.VP.A87E.01A |
|  | TCGA.M7.A723.01A | TCGA.HC.8258.01A |
|  | TCGA.EJ.5509.01A | TCGA.G9.6336.01A |
|  | TCGA.KC.A7F3.01A | TCGA.X4.A8KQ.01A |
|  | TCGA.CH.5737.01A | TCGA.HC.7210.01A |
|  | TCGA.G9.6377.01A | TCGA.HC.A6AQ.01A |
|  | TCGA.EJ.AB27.01A | TCGA.HC.A8CY.01A |
|  | TCGA.EJ.7782.01A | TCGA.G9.A9S4.01A |
|  | TCGA.EJ.5496.01A | TCGA.VP.A87H.01A |
|  | TCGA.EJ.A46G.01A | TCGA.YL.A9WI.01A |
|  | TCGA.EJ.A8FS.01A | TCGA.VP.A87D.01A |
|  | TCGA.EJ.7317.01A | TCGA.KK.A6E2.01A |
|  | TCGA.SU.A7E7.01A | TCGA.CH.5761.01A |
|  | TCGA.XJ.A83F.01A | TCGA.HC.7740.01A |
|  | TCGA.XQ.A8TA.01A | TCGA.XJ.A9DI.01A |
|  | TCGA.HC.A6AO.01A | TCGA.HC.7075.01A |
|  | TCGA.FC.A5OB.01A | TCGA.HC.A6HX.01A |
|  | TCGA.EJ.A46B.01A | TCGA.KK.A6DY.01A |
|  | TCGA.QU.A6IN.01A | TCGA.KK.A6E0.01A |
|  | TCGA.KK.A59Z.01A | TCGA.CH.5767.01A |
|  | TCGA.XK.AAJT.01A | TCGA.KK.A6E6.01A |
|  | TCGA.EJ.7125.01A | TCGA.YL.A9WY.01A |
|  | TCGA.J9.A52C.01A | TCGA.VN.A88N.01A |
|  | TCGA.YL.A8HL.01A | TCGA.YL.A9WK.01A |
|  | TCGA.ZG.A9LZ.01A | TCGA.2A.A8VL.01A |
|  | TCGA.J9.A8CP.01A | TCGA.V1.A9Z7.01A |
|  | TCGA.FC.A6HD.01A | TCGA.VP.AA1N.01A |
|  | TCGA.G9.6351.01A | TCGA.4L.AA1F.01A |
|  | TCGA.EJ.5532.01A | TCGA.EJ.5526.01A |
|  | TCGA.EJ.5525.01A | TCGA.HC.7742.01A |
|  | TCGA.ZG.A9L9.01A | TCGA.FC.A8O0.01A |
|  | TCGA.EJ.A65F.01A | TCGA.EJ.5508.01A |
|  | TCGA.2A.A8VX.01A | TCGA.HC.7749.01A |
|  | TCGA.KK.A8I4.01A | TCGA.V1.A9OT.01A |
|  | TCGA.KK.A8IA.01A | TCGA.CH.5741.01A |
|  | TCGA.Y6.A9XI.01A | TCGA.KK.A8IC.01A |
|  | TCGA.VN.A88I.01A | TCGA.YL.A9WH.01A |
|  | TCGA.M7.A71Z.01A | TCGA.ZG.A9L5.01A |
|  | TCGA.EJ.A46E.01A | TCGA.CH.5768.01A |
|  | TCGA.CH.5788.01A | TCGA.KC.A4BL.01A |
|  | TCGA.KK.A7AU.01A | TCGA.J4.A67K.01A |
|  | TCGA.G9.6333.01A | TCGA.G9.7525.01A |
|  | TCGA.EJ.7327.01A | TCGA.G9.6371.01A |
|  | TCGA.XK.AAIV.01A | TCGA.HC.A76W.01A |
|  | TCGA.M7.A720.01A | TCGA.EJ.A65G.01A |
|  | TCGA.V1.A9ZR.01A | TCGA.ZG.A9LS.01A |
|  | TCGA.EJ.8468.01A | TCGA.XJ.A9DK.01A |
|  | TCGA.ZG.A9N3.01A | TCGA.G9.6329.01A |
|  | TCGA.X4.A8KS.01A | TCGA.J9.A8CN.01A |
|  | TCGA.KK.A59V.01A | TCGA.V1.A9ZK.01A |
|  | TCGA.V1.A9OY.01A | TCGA.EJ.5542.01A |
|  | TCGA.QU.A6IP.01A | TCGA.V1.A8WS.01A |
|  | TCGA.HC.7077.01A | TCGA.ZG.A8QX.01A |
|  | TCGA.EJ.7123.01A | TCGA.YL.A8SC.01A |
|  | TCGA.EJ.5531.01A | TCGA.CH.5764.01A |
|  | TCGA.VN.A88K.01A | TCGA.CH.5789.01A |
|  | TCGA.VN.A88M.01A | TCGA.FC.A66V.01A |
|  | TCGA.EJ.5506.01A | TCGA.EJ.7330.01A |
|  | TCGA.XK.AAJU.01A | TCGA.HC.8258.01B |
|  | TCGA.YL.A8SI.01A | TCGA.YL.A8SL.01B |
|  | TCGA.G9.6499.01A | TCGA.XK.AAIW.01A |
|  | TCGA.QU.A6IL.01A | TCGA.G9.6385.01A |
|  | TCGA.J4.A67Q.01A | TCGA.HC.A76X.01A |
|  | TCGA.G9.6361.01A | TCGA.EJ.A46I.01A |
|  | TCGA.VN.A88L.01A | TCGA.2A.AAYO.01A |
|  | TCGA.KK.A8I9.01A | TCGA.2A.AAYF.01A |
|  | TCGA.V1.A9ZG.01A | TCGA.KK.A8I7.01A |
|  | TCGA.V1.A9OX.01A | TCGA.HC.A48F.01A |
|  | TCGA.G9.6373.01A | TCGA.YL.A9WJ.01A |
|  | TCGA.VP.A878.01A | TCGA.G9.6379.01A |
|  | TCGA.G9.6362.01A | TCGA.KK.A8II.01A |
|  | TCGA.HC.7819.01A | TCGA.G9.6353.01A |
|  | TCGA.KK.A6E8.01A | TCGA.HI.7170.01A |
|  | TCGA.EJ.A65E.01A | TCGA.EJ.A7NJ.01A |
|  | TCGA.ZG.A9L0.01A | TCGA.HC.A8D1.01A |
|  | TCGA.YL.A9WL.01A | TCGA.KK.A7B1.01A |
|  | TCGA.CH.5772.01A | TCGA.HC.A6HY.01A |
|  | TCGA.ZG.A9L2.01A | TCGA.V1.A9O7.01A |
|  | TCGA.HC.8266.01A | TCGA.XJ.A83H.01A |
|  | TCGA.KC.A7F6.01A |  |
| The accession number of RNA-sequencing profiles of 53 adjacent nontumor samples | TCGA.G9.6362.11A | TCGA.EJ.7317.11A |
|  | TCGA.G9.6342.11A | TCGA.HC.7737.11A |
|  | TCGA.HC.8260.11A | TCGA.HC.7747.11A |
|  | TCGA.EJ.7314.11A | TCGA.HC.7819.11A |
|  | TCGA.EJ.7784.11A | TCGA.HC.7752.11A |
|  | TCGA.EJ.7786.11A | TCGA.EJ.7789.11A |
|  | TCGA.EJ.7115.01A | TCGA.G9.6496.11A |
|  | TCGA.EJ.7123.11A | TCGA.EJ.7331.11A |
|  | TCGA.EJ.7328.11A | TCGA.HC.8262.11A |
|  | TCGA.HC.7742.11A | TCGA.EJ.A8FO.11A |
|  | TCGA.J4.A83J.11A | TCGA.HC.7738.11A |
|  | TCGA.HC.7211.11A | TCGA.EJ.7125.11A |
|  | TCGA.G9.6351.11A | TCGA.EJ.7785.11A |
|  | TCGA.CH.5761.11A | TCGA.EJ.7315.11A |
|  | TCGA.EJ.5511.01A | TCGA.EJ.7781.11A |
|  | TCGA.EJ.7782.11A | TCGA.EJ.7794.11A |
|  | TCGA.EJ.7115.11A | TCGA.HC.7745.11A |
|  | TCGA.G9.6499.11A | TCGA.CH.5769.11A |
|  | TCGA.EJ.7321.11A | TCGA.EJ.7792.11A |
|  | TCGA.G9.6365.11A | TCGA.EJ.7330.11A |
|  | TCGA.G9.6356.11A | TCGA.EJ.7797.11A |
|  | TCGA.EJ.7327.11A | TCGA.G9.6348.11A |
|  | TCGA.HC.8258.11A | TCGA.HC.7740.11A |
|  | TCGA.G9.6333.11A | TCGA.EJ.7793.11A |
|  | TCGA.G9.6384.11A | TCGA.G9.6363.11A |
|  | TCGA.CH.5768.11A | TCGA.EJ.7783.11A |
|  | TCGA.HC.8259.11A | |

**Table S2. (Another page)**

**Table S3.** 100 DNAm-derived differentially expressed gene**.**

| **gene** | **normalMean** | **TumorMean** | **logFC** | **pValue** | **adjustP** | **cor** | **corPavlue** |
| --- | --- | --- | --- | --- | --- | --- | --- |
| ETNK2 | 0.367471 | 0.468357 | 0.349976 | 1.37E-24 | 1.46E-22 | -0.45401 | 1.39E-21 |
| B3GNT8 | 0.40298 | 0.552536 | 0.455362 | 3.24E-24 | 3.44E-22 | -0.54409 | 5.70E-32 |
| HIF3A | 0.465082 | 0.690076 | 0.56927 | 4.65E-24 | 4.93E-22 | -0.42857 | 3.63E-19 |
| APOBEC3C | 0.082913 | 0.295407 | 1.83303 | 4.82E-24 | 5.11E-22 | -0.62051 | 1.28E-43 |
| CXCL6 | 0.182676 | 0.354543 | 0.956675 | 5.30E-24 | 5.62E-22 | -0.42321 | 1.11E-18 |
| EFS | 0.180806 | 0.411739 | 1.187288 | 7.15E-24 | 7.58E-22 | -0.60231 | 1.48E-40 |
| AOX1 | 0.223477 | 0.552866 | 1.306806 | 1.55E-23 | 1.64E-21 | -0.58567 | 6.33E-38 |
| GSTP1 | 0.26571 | 0.507298 | 0.93298 | 1.55E-23 | 1.64E-21 | -0.62237 | 6.07E-44 |
| DUOX1 | 0.358119 | 0.527703 | 0.559287 | 1.81E-23 | 1.92E-21 | -0.40818 | 2.26E-17 |
| CHST3 | 0.467172 | 0.576393 | 0.303101 | 3.38E-23 | 3.59E-21 | -0.45919 | 4.22E-22 |
| HLF | 0.226492 | 0.406004 | 0.842033 | 4.99E-23 | 5.28E-21 | -0.32996 | 1.55E-11 |
| CTF1 | 0.167919 | 0.321896 | 0.938824 | 6.01E-23 | 6.37E-21 | -0.62147 | 8.73E-44 |
| GSTM2 | 0.234972 | 0.438578 | 0.900343 | 6.38E-23 | 6.76E-21 | -0.47703 | 5.95E-24 |
| GJA1 | 0.358298 | 0.478481 | 0.417302 | 8.15E-23 | 8.64E-21 | -0.38853 | 9.37E-16 |
| GATA3 | 0.488208 | 0.686587 | 0.491946 | 8.44E-23 | 8.94E-21 | -0.5766 | 1.49E-36 |
| CCK | 0.155489 | 0.262805 | 0.757177 | 9.16E-23 | 9.70E-21 | -0.36155 | 1.05E-13 |
| JPH2 | 0.373059 | 0.485883 | 0.381204 | 1.07E-22 | 1.13E-20 | -0.39496 | 2.85E-16 |
| AKR1B1 | 0.188882 | 0.416225 | 1.13988 | 1.41E-22 | 1.49E-20 | -0.40725 | 2.71E-17 |
| ANXA2 | 0.300514 | 0.472352 | 0.652431 | 1.99E-22 | 2.11E-20 | -0.54116 | 1.40E-31 |
| CCDC8 | 0.405226 | 0.582343 | 0.523144 | 2.09E-22 | 2.21E-20 | -0.55438 | 2.29E-33 |
| CRYAB | 0.21109 | 0.419654 | 0.991339 | 2.85E-22 | 3.02E-20 | -0.37598 | 8.90E-15 |
| ACOX2 | 0.532598 | 0.659992 | 0.309401 | 3.89E-22 | 4.12E-20 | -0.48924 | 2.78E-25 |
| FBXO17 | 0.372394 | 0.47974 | 0.365425 | 6.08E-22 | 6.44E-20 | -0.66518 | 4.65E-52 |
| C2orf88 | 0.456657 | 0.64126 | 0.489799 | 8.18E-22 | 8.67E-20 | -0.49559 | 5.38E-26 |
| HOXA7 | 0.279171 | 0.586145 | 1.070109 | 9.59E-22 | 1.02E-19 | -0.48062 | 2.45E-24 |
| KIAA1614 | 0.475934 | 0.582818 | 0.292283 | 1.09E-21 | 1.15E-19 | -0.36505 | 5.85E-14 |
| CPXM1 | 0.180384 | 0.375862 | 1.059133 | 1.14E-21 | 1.21E-19 | -0.34814 | 9.38E-13 |
| ACSS3 | 0.222059 | 0.502722 | 1.178821 | 1.32E-21 | 1.40E-19 | -0.55542 | 1.64E-33 |
| ACSM1 | 0.831816 | 0.614082 | -0.43783 | 1.58E-21 | 1.67E-19 | -0.74118 | 2.32E-70 |
| CPA6 | 0.411228 | 0.58291 | 0.503335 | 1.79E-21 | 1.90E-19 | -0.50947 | 1.31E-27 |
| LTF | 0.360383 | 0.59075 | 0.713017 | 2.12E-21 | 2.25E-19 | -0.49868 | 2.39E-26 |
| FAM110C | 0.169934 | 0.284666 | 0.744294 | 2.78E-21 | 2.94E-19 | -0.42245 | 1.29E-18 |
| ACSF2 | 0.420208 | 0.589177 | 0.487599 | 3.00E-21 | 3.18E-19 | -0.37353 | 1.37E-14 |
| HAAO | 0.2556 | 0.521864 | 1.029786 | 5.09E-21 | 5.39E-19 | -0.64552 | 3.59E-48 |
| ITPRIPL1 | 0.380779 | 0.525461 | 0.464632 | 5.20E-21 | 5.51E-19 | -0.46608 | 8.39E-23 |
| GPR87 | 0.522307 | 0.691265 | 0.404342 | 5.75E-21 | 6.10E-19 | -0.51636 | 1.94E-28 |
| GPX3 | 0.265617 | 0.462297 | 0.799474 | 7.77E-21 | 8.24E-19 | -0.4394 | 3.60E-20 |
| CNN1 | 0.440528 | 0.551394 | 0.32385 | 1.35E-20 | 1.43E-18 | -0.35253 | 4.64E-13 |
| KRT23 | 0.503299 | 0.632536 | 0.329732 | 3.41E-20 | 3.61E-18 | -0.51809 | 1.20E-28 |
| CAPG | 0.531943 | 0.696257 | 0.388349 | 4.58E-20 | 4.85E-18 | -0.45242 | 2.00E-21 |
| DUOX2 | 0.311276 | 0.459932 | 0.563223 | 9.68E-20 | 1.03E-17 | -0.31972 | 6.93E-11 |
| GALR1 | 0.19955 | 0.347659 | 0.800924 | 1.68E-19 | 1.78E-17 | -0.33962 | 3.57E-12 |
| GPX2 | 0.5557 | 0.747656 | 0.428069 | 1.79E-19 | 1.89E-17 | -0.64243 | 1.39E-47 |
| ANGPTL1 | 0.68668 | 0.819426 | 0.254976 | 3.43E-19 | 3.64E-17 | -0.37346 | 1.38E-14 |
| CCNI2 | 0.354944 | 0.54579 | 0.620751 | 7.52E-19 | 7.97E-17 | -0.39308 | 4.05E-16 |
| LAMB3 | 0.471686 | 0.58919 | 0.320907 | 7.92E-19 | 8.40E-17 | -0.65379 | 9.05E-50 |
| CPA1 | 0.565659 | 0.734799 | 0.377417 | 1.06E-18 | 1.13E-16 | -0.39116 | 5.77E-16 |
| DEFB1 | 0.425641 | 0.567345 | 0.414588 | 1.07E-18 | 1.14E-16 | -0.41248 | 9.68E-18 |
| DPYS | 0.289055 | 0.450953 | 0.641632 | 2.50E-18 | 2.66E-16 | -0.47588 | 7.89E-24 |
| DNAH5 | 0.682187 | 0.566525 | -0.26803 | 2.64E-18 | 2.80E-16 | -0.53361 | 1.35E-30 |
| FADS2 | 0.130956 | 0.311095 | 1.248278 | 3.15E-18 | 3.34E-16 | -0.55594 | 1.39E-33 |
| BVES | 0.420135 | 0.547326 | 0.381546 | 3.25E-18 | 3.44E-16 | -0.31883 | 7.87E-11 |
| CYP27A1 | 0.257076 | 0.520665 | 1.018163 | 1.02E-17 | 1.08E-15 | -0.77594 | 4.71E-81 |
| GDF15 | 0.299843 | 0.236503 | -0.34235 | 1.17E-17 | 1.24E-15 | -0.34777 | 9.95E-13 |
| LGALS7B | 0.613 | 0.715291 | 0.222643 | 1.20E-17 | 1.27E-15 | -0.41433 | 6.71E-18 |
| ALDH1A3 | 0.425716 | 0.304352 | -0.48415 | 1.23E-17 | 1.31E-15 | -0.60861 | 1.35E-41 |
| GIPC2 | 0.290573 | 0.496791 | 0.773736 | 1.85E-17 | 1.96E-15 | -0.37601 | 8.85E-15 |
| ALDH3A1 | 0.294199 | 0.400737 | 0.445865 | 2.22E-17 | 2.35E-15 | -0.53002 | 3.89E-30 |
| HFE | 0.29498 | 0.518722 | 0.814345 | 2.36E-17 | 2.50E-15 | -0.57383 | 3.84E-36 |
| ITGB8 | 0.178997 | 0.227803 | 0.347849 | 4.27E-17 | 4.52E-15 | -0.33053 | 1.42E-11 |
| CHAD | 0.276553 | 0.514007 | 0.894233 | 5.87E-17 | 6.23E-15 | -0.35809 | 1.87E-13 |
| EVX2 | 0.225069 | 0.404778 | 0.846764 | 7.84E-17 | 8.31E-15 | -0.45921 | 4.20E-22 |
| GSTM4 | 0.202914 | 0.250691 | 0.305042 | 1.32E-16 | 1.40E-14 | -0.5077 | 2.12E-27 |
| B3GNT9 | 0.287009 | 0.403789 | 0.492505 | 1.46E-16 | 1.55E-14 | -0.43299 | 1.43E-19 |
| HOXD11 | 0.16618 | 0.284211 | 0.774218 | 3.77E-16 | 4.00E-14 | -0.36544 | 5.48E-14 |
| LRRC4 | 0.34085 | 0.511208 | 0.584773 | 9.89E-16 | 1.05E-13 | -0.48459 | 9.05E-25 |
| ASS1 | 0.257537 | 0.292181 | 0.182082 | 2.73E-15 | 2.90E-13 | -0.31655 | 1.09E-10 |
| CDO1 | 0.237503 | 0.434631 | 0.871843 | 3.59E-15 | 3.81E-13 | -0.58996 | 1.37E-38 |
| CLDN8 | 0.557493 | 0.379676 | -0.55419 | 5.89E-15 | 6.25E-13 | -0.57458 | 2.97E-36 |
| CCDC69 | 0.186826 | 0.203714 | 0.124847 | 7.44E-15 | 7.88E-13 | -0.35477 | 3.22E-13 |
| FUT2 | 0.380223 | 0.482723 | 0.344348 | 7.44E-15 | 7.88E-13 | -0.33751 | 4.94E-12 |
| COL9A2 | 0.22038 | 0.256043 | 0.21639 | 7.65E-15 | 8.11E-13 | -0.35336 | 4.06E-13 |
| KRT15 | 0.516742 | 0.646396 | 0.322973 | 8.55E-15 | 9.06E-13 | -0.57549 | 2.18E-36 |
| LY6G6D | 0.414506 | 0.527089 | 0.346656 | 2.07E-14 | 2.19E-12 | -0.3903 | 6.77E-16 |
| KRT7 | 0.394966 | 0.615434 | 0.639875 | 2.31E-14 | 2.44E-12 | -0.44452 | 1.17E-20 |
| CD40 | 0.337803 | 0.502811 | 0.573835 | 2.39E-14 | 2.53E-12 | -0.51551 | 2.47E-28 |
| B3GALT6 | 0.857726 | 0.721676 | -0.24917 | 3.26E-14 | 3.45E-12 | -0.35271 | 4.51E-13 |
| HOXD13 | 0.341445 | 0.448798 | 0.39441 | 6.01E-14 | 6.37E-12 | -0.41955 | 2.34E-18 |
| IL1RL2 | 0.437916 | 0.599916 | 0.454106 | 1.25E-13 | 1.32E-11 | -0.79276 | 5.99E-87 |
| CPNE6 | 0.585086 | 0.686034 | 0.22963 | 2.93E-13 | 3.11E-11 | -0.33665 | 5.63E-12 |
| KRT5 | 0.416255 | 0.55847 | 0.424012 | 1.15E-12 | 1.22E-10 | -0.39432 | 3.21E-16 |
| CD38 | 0.331343 | 0.484561 | 0.548352 | 2.66E-12 | 2.82E-10 | -0.77197 | 9.77E-80 |
| GSTM5 | 0.506526 | 0.627407 | 0.308765 | 4.01E-12 | 4.25E-10 | -0.32839 | 1.95E-11 |
| CALML3 | 0.569896 | 0.695664 | 0.287693 | 4.11E-12 | 4.36E-10 | -0.67875 | 6.39E-55 |
| EID3 | 0.261828 | 0.404116 | 0.626149 | 2.01E-11 | 2.13E-09 | -0.66787 | 1.30E-52 |
| ENTPD3 | 0.188673 | 0.232055 | 0.298583 | 4.42E-11 | 4.69E-09 | -0.42292 | 1.17E-18 |
| CHRM5 | 0.618725 | 0.556999 | -0.15162 | 1.94E-10 | 2.06E-08 | -0.33046 | 1.44E-11 |
| ELL3 | 0.101969 | 0.093085 | -0.13151 | 2.65E-10 | 2.81E-08 | -0.34486 | 1.58E-12 |
| ID1 | 0.085755 | 0.146347 | 0.771095 | 6.02E-09 | 6.38E-07 | -0.38939 | 8.00E-16 |
| FABP5 | 0.243437 | 0.298271 | 0.293077 | 1.04E-08 | 1.11E-06 | -0.39136 | 5.56E-16 |
| C2CD4A | 0.146491 | 0.232412 | 0.665875 | 3.46E-08 | 3.66E-06 | -0.35626 | 2.53E-13 |
| GALNT5 | 0.251899 | 0.326018 | 0.372103 | 6.74E-08 | 7.14E-06 | -0.50467 | 4.83E-27 |
| F5 | 0.204349 | 0.186326 | -0.13321 | 7.53E-08 | 7.98E-06 | -0.3496 | 7.43E-13 |
| KCTD14 | 0.550151 | 0.608194 | 0.144704 | 9.89E-08 | 1.05E-05 | -0.5773 | 1.17E-36 |
| CDKL2 | 0.227376 | 0.340685 | 0.583357 | 1.26E-07 | 1.34E-05 | -0.44763 | 5.86E-21 |
| CCL18 | 0.710527 | 0.644638 | -0.1404 | 1.31E-07 | 1.39E-05 | -0.32209 | 4.92E-11 |
| GSTM1 | 0.32222 | 0.572258 | 0.82862 | 2.02E-07 | 2.15E-05 | -0.70204 | 3.30E-60 |
| HNF1A | 0.558079 | 0.604069 | 0.114243 | 2.80E-05 | 0.002968 | -0.7105 | 2.95E-62 |
| HABP2 | 0.585511 | 0.637639 | 0.123044 | 3.10E-05 | 0.003291 | -0.60532 | 4.75E-41 |
| KRT13 | 0.674404 | 0.723116 | 0.100614 | 0.000154 | 0.016368 | -0.3485 | 8.86E-13 |

**Table S5. The accession number of the sample set from GEO**

| The accession number of RNA-sequencing profiles of 139 PCA samples from GSE21034 | ID |  |
| --- | --- | --- |
|  | GSM527958 | GSM527865 |
|  | GSM528013 | GSM527974 |
|  | GSM527866 | GSM527944 |
|  | GSM527909 | GSM527943 |
|  | GSM527895 | GSM527934 |
|  | GSM527950 | GSM527871 |
|  | GSM527938 | GSM527911 |
|  | GSM527915 | GSM527859 |
|  | GSM527920 | GSM527894 |
|  | GSM527979 | GSM527863 |
|  | GSM527942 | GSM527963 |
|  | GSM527961 | GSM527876 |
|  | GSM527928 | GSM527948 |
|  | GSM527988 | GSM527908 |
|  | GSM527970 | GSM527959 |
|  | GSM527860 | GSM527953 |
|  | GSM527927 | GSM527957 |
|  | GSM527968 | GSM527893 |
|  | GSM527907 | GSM527951 |
|  | GSM527989 | GSM527889 |
|  | GSM527932 | GSM527885 |
|  | GSM527923 | GSM527995 |
|  | GSM527992 | GSM527987 |
|  | GSM527937 | GSM527906 |
|  | GSM527918 | GSM528008 |
|  | GSM527896 | GSM527917 |
|  | GSM527910 | GSM527956 |
|  | GSM527919 | GSM527998 |
|  | GSM527891 | GSM527965 |
|  | GSM527945 | GSM527980 |
|  | GSM527931 | GSM527926 |
|  | GSM527985 | GSM527960 |
|  | GSM527902 | GSM527874 |
|  | GSM527933 | GSM527947 |
|  | GSM527900 | GSM527912 |
|  | GSM527901 | GSM527867 |
|  | GSM527935 | GSM527993 |
|  | GSM527941 | GSM527862 |
|  | GSM527883 | GSM528005 |
|  | GSM527916 | GSM528006 |
|  | GSM527973 | GSM527904 |
|  | GSM527903 | GSM527984 |
|  | GSM527899 | GSM527873 |
|  | GSM527858 | GSM527868 |
|  | GSM527875 | GSM527972 |
|  | GSM527870 | GSM527952 |
|  | GSM527929 | GSM527888 |
|  | GSM527913 | GSM527869 |
|  | GSM527921 | GSM527878 |
|  | GSM527977 | GSM527887 |
|  | GSM527914 | GSM527886 |
|  | GSM527966 | GSM527955 |
|  | GSM527922 | GSM527861 |
|  | GSM527949 | GSM527864 |
|  | GSM527936 | GSM527884 |
|  | GSM527879 | GSM527881 |
|  | GSM527897 | GSM527962 |
|  | GSM527924 | GSM527872 |
|  | GSM527892 | GSM527971 |
|  | GSM527877 | GSM527882 |
|  | GSM527898 | GSM527940 |
|  | GSM527975 | GSM528011 |
|  | GSM527991 | GSM527982 |
|  | GSM527930 | GSM527880 |
|  | GSM527939 | GSM527954 |
|  | GSM527994 | GSM528007 |
|  | GSM527905 | GSM528014 |
|  | GSM527925 | GSM527890 |
|  | GSM527990 | GSM527946 |

**Table S6. Primers for real time-PCR**

| CCK-F1 | TGAGGGTATCGCAGAGAAC | 159bp |
| --- | --- | --- |
| CCK-R1 | AGTCCCGGTCACTTATCCT |  |
| CD38-F1 | CATGGTGTGGTGAATTCAAC | 161bp |
| CD38-R1 | GGATCCATTGAGCATCACAT |  |
| CYP27A1-F1 | CTGGCTACCTGCACTTCTTAC | 150bp |
| CYP27A1-R1 | GGATCTCAGGGTCCTTTGAG |  |
| EID3-F1 | CCTGGATGGTAAAAGCTGAGA | 150bp |
| EID3-R1 | GGTCCAACTTCTGCAACTTTG |  |
| HABP2-F1 | GGCGATGGCTACTCTTAC | 219bp |
| HABP2-R1 | TTCCCATTTCACCTTGTC |  |
| LRRC4-F1 | ATGTCCTCCGTGAAGTGGTT | 150bp |
| LRRC4-R1 | ATTGGTCACCATGCATGTGT |  |
| LY6G6D-F1 | GGTGGGAGACGTGACTTATC | 140bp |
| LY6G6D-R1 | ACAGTCCTGGCAAGAGACA |  |
| GAPDH-F1 | GGGAAACTGTGGCGTGAT | 299bp |
| GAPDH-R1 | GAGTGGGTGTCGCTGTTGA |  |

**Table S7. Seven gene expression of prostate cancer cell line from CCLE database (CCLE RNAseq gene expression data fpr 1019 cell lines (read counts)).**

| **ID** | **gene_symbol** | **22Rv1** | **DU 145** | **LNCaP clone FGC** | **MDA PCa 2b** | **NCI-H660** | **PC-3** | **VCaP** |
| --- | --- | --- | --- | --- | --- | --- | --- | --- |
| 885_at | CCK | 4.0277 | 3.7523 | 3.8071 | 4.3591 | 3.7521 | 4.0341 | 3.9826 |
| 952_at | CD38 | 4.4827 | 4.3176 | 4.7755 | 4.9426 | 4.9271 | 4.848 | 4.6131 |
| 1593_at | CYP27A1 | 5.6343 | 6.1612 | 5.568 | 5.8609 | 6.0411 | 6.4956 | 5.8202 |
| 493861_at | EID3 | 5.1016 | 8.8683 | 5.0973 | 5.7753 | 4.628 | 5.3086 | 6.2717 |
| 3026_at | HABP2 | 4.5232 | 3.6814 | 3.7256 | 3.8659 | 3.6556 | 3.6545 | 4.119 |
| 64101_at | LRRC4 | 4.5642 | 4.6408 | 4.5148 | 4.8361 | 5.6115 | 4.6695 | 4.7391 |
| 58530_at | LY6G6D | 3.7681 | 3.6786 | 4.0033 | 4.0251 | 3.7812 | 4.0892 | 4.0717 |

**Table S8. Seven gene expression of prostate cancer cell line from GSE21034**

| **gene_symbol** | **DU145** | **LNCAP** | **PC3** | **Vcap** |
| --- | --- | --- | --- | --- |
| CCK | 190.0492 | 152.144 | 144.5833 | 137.207 |
| CD38 | 150.798 | 111.469 | 102.0341 | 115.0706 |
| CYP27A1 | 157.6865 | 87.22201 | 160.123 | 94.36324 |
| EID3 | 144.8181 | 99.36902 | 93.05987 | 192.2534 |
| HABP2 | 89.37865 | 85.85694 | 84.30126 | 102.4057 |
| LRRC4 | 107.5404 | 97.57386 | 84.15559 | 96.10403 |
| LY6G6D | 176.8861 | 166.8663 | 172.809 | 171.6124 |

**Supplement file of Data standardization analysis**

The gene expression of the four DMGs were scaled to a reasonable range using the
following equations: CPM in the risk score formula represents data normalization based on the R package ‘edgeR’.

Library(edgeR)

dge = DGEList(counts = RNA Count value).

dge <-calcNormFactors(dge).

CPM <- cpm(dge, log = TRUE, prior.count = 3).
