# Supplementary material for: Clinical and Biological Significance of DNA Methylation-Driven Differentially Expressed Genes in Biochemical Recurrence After Radical Prostatectomy
Source: Front Genet. 2022 Feb 2;13:727307. doi: 10.3389/fgene.2022.727307 (PMC8847683; doi:10.3389/fgene.2022.727307)
Supplement: Supplementary file 4 [file Table3.DOCX]

rm(list = ls())

memory.limit(size=1000000)

setwd("D:\\3_bioinformatical analysis\\methylation-DNA\\1A_new_analysis")

1.

pd.all <- read.delim("sample.tsv", header = T, stringsAsFactors = F)

dim(pd.all)

colnames(pd.all)

pd <- pd.all[,c("case_id","case_submitter_id","sample_submitter_id","sample_type")]

table(pd$sample_type)

tissue <- c("Primary Tumor","Solid Tissue Normal")

pd_tissue <- pd[pd$sample_type %in% tissue,]

table(pd_tissue$sample_type)

table(pd_tissue$case_submitter_id)

deleteuniquelines <- function(x){

stand.col <- x$case_submitter_id

count <- table(stand.col)

if(all(count < 2)) stop("no repeated records")

else {

ind <- sapply(stand.col, function(t) ifelse(count[as.character(t)] >1, TRUE, FALSE))

}

return(x[ind,])

}

pd_tissue_filtered <- deleteuniquelines(pd_tissue)

dim(pd_tissue_filtered)

nt <- pd_tissue_filtered[pd_tissue_filtered$sample_type == "Solid Tissue Normal",]

tt <- pd_tissue_filtered[pd_tissue_filtered$sample_type == "Primary Tumor",]

#只取samle_submitter_id最后一位是A的样品

tt<- tt[substr(tt$sample_submitter_id,16,16) == "A",]

tt <- tt[tt$case_submitter_id %in% nt$case_submitter_id,]

dim(tt)

paired_tissue <- rbind(nt,tt)

dim(paired_tissue)

tumor_site <- read.delim("clinical.tsv", header = T, stringsAsFactors = F)

tumor_site <- tumor_site[,c("case_id","case_submitter_id","tissue_or_organ_of_origin")]

library(stringr)

tumor_site[,c("anatomic_neoplasm_subdivision", "takeout")] <- str_split_fixed(tumor_site$tissue_or_organ_of_origin,",",2)

tumor_site <- tumor_site[,c(1,2,4)]

table(tumor_site$anatomic_neoplasm_subdivision)

oscc <- c("Prostate gland")

tumor_site <- tumor_site[tumor_site$anatomic_neoplasm_subdivision %in% oscc,]

dim(tumor_site)

tumor_site_unique <- unique(tumor_site$case_submitter_id)

tumor_site_unique <- as.data.frame(tumor_site_unique)

colnames(tumor_site_unique) <- "case_submitter_id"

merge_info <- paired_tissue[paired_tissue$case_submitter_id %in% tumor_site_unique$case_submitter_id,]

dim(merge_info)

methy_data <- data.table::fread("TCGA-PRAD.methylation450.tsv", data.table = F)

methy_data[1:4,1:4]

head(merge_info)[1:4,1:4]

# merge_info$sample_submitter_id <- substr(merge_info$sample_submitter_id,1,15)

head(merge_info)

write.table(merge_info$sample_submitter_id, file = "paired_sample_name_from_clinical.txt",quote = F, row.names = F, col.names = F)

methy_data[1:4,1:6]

nt_paired <- merge_info[merge_info$sample_type == "Solid Tissue Normal",] #只有50个匹配

tt_paired <- merge_info[merge_info$sample_type == "Primary Tumor",]

methy_nt <- methy_data[,colnames(methy_data) %in% nt_paired$sample_submitter_id]

methy_tt <- methy_data[,colnames(methy_data) %in% tt_paired$sample_submitter_id]

methy_combine <- cbind(methy_nt,methy_tt)

table(colnames(methy_combine))

#############################################

head(methy_combine)

table(colnames(methy_paired))

rownames(methy_paired) <- methy_data$`Composite Element REF`

write.csv(methy_paired, file = "OSCC_168paired_methydata.csv")

methy <- data.table::fread("OSCC_168paired_methydata.csv", data.table = F)

2.

library(ChAMP)

library(dplyr)

library(tibble)

colnames(methy)

merge_info$sample_submitter_id

methy_sort <- methy[,c("V1","TCGA-G9-6364-11A", "TCGA-CH-5761-11A", "TCGA-EJ-7317-11A", "TCGA-CH-5768-11A",

"TCGA-EJ-7784-11A", "TCGA-EJ-7125-11A", "TCGA-G9-6356-11A", "TCGA-G9-6348-11A", "TCGA-G9-6365-11A",

"TCGA-G9-6496-11A", "TCGA-CH-5772-11A", "TCGA-HC-7752-11A", "TCGA-HC-7819-11A", "TCGA-G9-6353-11A",

"TCGA-CH-5771-11A", "TCGA-CH-5766-11A", "TCGA-G9-6367-11A", "TCGA-CH-5769-11A", "TCGA-EJ-7792-11A",

"TCGA-G9-6373-11A", "TCGA-G9-6384-11A", "TCGA-HC-7737-11A", "TCGA-EJ-7123-11A", "TCGA-G9-6494-11A",

"TCGA-EJ-7781-11A", "TCGA-CH-5765-11A", "TCGA-EJ-7794-11A", "TCGA-HC-7211-11A", "TCGA-CH-5767-11B",

"TCGA-EJ-7782-11A", "TCGA-CH-5763-11A", "TCGA-EJ-7331-11A", "TCGA-G9-6332-11A", "TCGA-G9-6499-11A",

"TCGA-EJ-7786-11A", "TCGA-G9-6362-11A", "TCGA-G9-6351-11A", "TCGA-HC-7745-11A", "TCGA-EJ-7328-11A",

"TCGA-EJ-7327-11A", "TCGA-G9-6363-11A", "TCGA-HC-7820-11A", "TCGA-G9-6385-11A", "TCGA-G9-6342-11A",

"TCGA-EJ-7789-11A", "TCGA-EJ-7785-11A", "TCGA-G9-6333-11A", "TCGA-CH-5762-11A", "TCGA-CH-5764-11A",

"TCGA-HC-7742-11A", "TCGA-KK-A8IA-01A", "TCGA-KK-A6E1-01A", "TCGA-EJ-7797-01A", "TCGA-HC-7738-01A",

"TCGA-G9-6356-01A", "TCGA-EJ-7125-01A", "TCGA-EJ-7789-01A", "TCGA-G9-6496-01A", "TCGA-G9-6348-01A",

"TCGA-KK-A7B1-01A", "TCGA-HC-7752-01A", "TCGA-G9-6362-01A", "TCGA-KK-A7B2-01A", "TCGA-HC-7819-01A",

"TCGA-KK-A7AW-01A", "TCGA-KK-A7B3-01A", "TCGA-EJ-7782-01A", "TCGA-EJ-7328-01A", "TCGA-KK-A8IK-01A",

"TCGA-CH-5767-01A", "TCGA-HC-8261-01A", "TCGA-EJ-7314-01A", "TCGA-EJ-7317-01A", "TCGA-EJ-8468-01A",

"TCGA-HC-7737-01A", "TCGA-G9-6499-01A", "TCGA-CH-5764-01A", "TCGA-HC-7742-01A", "TCGA-HC-8259-01A",

"TCGA-J4-A83J-01A", "TCGA-HC-8258-01A", "TCGA-EJ-7792-01A", "TCGA-EJ-7784-01A", "TCGA-KK-A6E6-01A",

"TCGA-EJ-8472-01A", "TCGA-EJ-7123-01A", "TCGA-EJ-7330-01A", "TCGA-KK-A8I4-01A", "TCGA-KK-A8I6-01A",

"TCGA-CH-5769-01A", "TCGA-KK-A7AQ-01A", "TCGA-KK-A59Z-01A", "TCGA-KK-A8I8-01A", "TCGA-CH-5761-01A",

"TCGA-KK-A6E2-01A", "TCGA-HC-8262-01A", "TCGA-KK-A8I7-01A", "TCGA-EJ-7321-01A", "TCGA-KK-A7AP-01A",

"TCGA-KK-A6E0-01A", "TCGA-KK-A6DY-01A", "TCGA-KK-A5A1-01A", "TCGA-KK-A59V-01A", "TCGA-KK-A7AV-01A",

"TCGA-KK-A6E4-01A", "TCGA-G9-6494-01A", "TCGA-KK-A8IG-01A", "TCGA-G9-6373-01A", "TCGA-CH-5771-01A",

"TCGA-EJ-7793-01A", "TCGA-EJ-7327-01A", "TCGA-KK-A7B4-01A", "TCGA-G9-6351-01A", "TCGA-KK-A8IL-01A",

"TCGA-HC-7211-01A", "TCGA-EJ-7331-01A", "TCGA-KK-A8IJ-01A", "TCGA-CH-5762-01A", "TCGA-KK-A8I5-01A",

"TCGA-EJ-7783-01A", "TCGA-G9-6367-01A", "TCGA-G9-6363-01A", "TCGA-KK-A6E8-01A", "TCGA-KK-A59X-01A",

"TCGA-HC-8265-01A", "TCGA-EJ-7785-01A", "TCGA-G9-6384-01A", "TCGA-EJ-7315-01A", "TCGA-KK-A8II-01A",

"TCGA-KK-A7B0-01A", "TCGA-G9-6333-01A", "TCGA-KK-A59Y-01A", "TCGA-KK-A8I9-01A", "TCGA-EJ-7786-01A",

"TCGA-G9-6342-01A", "TCGA-CH-5765-01A", "TCGA-KK-A8ID-01A", "TCGA-EJ-7781-01A", "TCGA-G9-6353-01A",

"TCGA-KK-A6E3-01A", "TCGA-KK-A8IM-01A", "TCGA-CH-5763-01A", "TCGA-EJ-A8FO-01A", "TCGA-G9-6385-01A",

"TCGA-HC-8256-01A", "TCGA-KK-A7AY-01A", "TCGA-CH-5768-01A", "TCGA-KK-A8IB-01A", "TCGA-KK-A6E7-01A",

"TCGA-CH-5772-01A", "TCGA-CH-5766-01A", "TCGA-KK-A8IH-01A", "TCGA-EJ-7794-01A", "TCGA-EJ-8474-01A",

"TCGA-G9-6332-01A", "TCGA-KK-A6E5-01A", "TCGA-G9-6365-01A", "TCGA-KK-A8IC-01A", "TCGA-EJ-7115-01A",

"TCGA-KK-A7AZ-01A", "TCGA-HC-7745-01A", "TCGA-G9-6364-01A", "TCGA-KK-A8IF-01A", "TCGA-KK-A7AU-01A",

"TCGA-HC-7747-01A", "TCGA-HC-8260-01A", "TCGA-HC-7740-01A", "TCGA-HC-7820-01A")]

a <- column_to_rownames(methy_sort,"V1")

beta_value <- as.matrix(a)

library(impute)

beta <- impute.knn(beta_value)

#beta delete NA

sum(is.na(beta))

beta <- beta$data

beta <- beta+0.00001

#ready pd document

pd_1 <- as.data.frame(colnames(beta))

pd_info <- merge_info[merge_info$sample_submitter_id %in% pd_1$`colnames(beta)`,]

colnames(pd_1) <- "sample_submitter_id"

pd <- merge(pd_1,pd_info, by="sample_submitter_id", all.x=TRUE)

#chAM filtering

myLoad <- champ.filter(beta = beta, pd = pd)

dim(myLoad$beta)

save(myLoad$beta, file = "PRAD_449_methydata_ChAMPfiltered.Rdata")
